# Occurrence of Antimicrobial Resistance in the Environment in Germany, Austria, and Switzerland: A Narrative Review of Existing Evidence

**DOI:** 10.3390/microorganisms10040728

**Published:** 2022-03-29

**Authors:** Marina Treskova, Alexander Kuhlmann, Fritjof Freise, Lothar Kreienbrock, Sandra Brogden

**Affiliations:** 1 Department of Biometry, Epidemiology and Information Processing, University of Veterinary Medicine Hannover, 30559 Hannover, Germany; marina.treskova@uni-heidelberg.de (M.T.); fritjof.freise@tiho-hannover.de (F.F.); lothar.kreienbrock@tiho-hannover.de (L.K.); 2 Heidelberg Institute of Global Health, Faculty of Medicine, University Heidelberg, 69120 Heidelberg, Germany; 3 Faculty of Medicine, Martin Luther University of Halle Wittenberg, 06108 Halle (Saale), Germany; alexander.kuhlmann@uk-halle.de

**Keywords:** multidrug resistance, antibiotic resistance genes (ARGs), antibiotic-resistant bacteria (ARB), One Health approach, environment, wastewater treatment plants, water, sediment, soil, wildlife, animal husbandry

## Abstract

(1) Background: This study summarizes the current research on antibiotic resistance (AR) in the environment conducted in Austria, Germany, and Switzerland; (2) Methods: A narrative systematic literature review of epidemiological studies based on searches in EMBASE and CAB abstracts (up to 16 June2021) was conducted. Environmental reservoirs included water sources, wastewater, animal husbandry, wildlife, soil, and sediment; (3) Results: Four hundred and four records were screened, and 52 studies were included. Thirteen studies examined aquatic environments, and eleven investigated wastewater. Eight studies investigated both wildlife and animal husbandry. Less evidence was available for sediments, soil, and air. Considerable heterogeneity in research focus, study design, sampling, and measurement of resistance was observed. Resistance to all categories of antimicrobials in the WHO CIA list was identified. Resistance to critically important and highly important substances was reported most frequently; (4) Conclusions: The current research scope presents data-gathering efforts. Usage of a unified protocol for isolate collection, selecting sampling sites, and susceptibility testing is required to provide results that can be compared between the studies and reservoirs. Epidemiological, environmental, and ecological factors should be considered in surveys of the environmental dissemination of AR. Systematic epidemiological studies investigating AR at the interface of human, animal, and environmental health are needed.

## 1. Introduction

Antimicrobial resistance has been recognized as one of the major current global health challenges. In Europe in 2015, 6.44 (5.54–7.48)/100,000 deaths were estimated to be caused by infections with antibiotic-resistant bacteria [1]. Worldwide, a recent systematic analysis of the global burden of antimicrobial resistance estimated 4.95 million (3.62–6.57) deaths associated with resistant bacteria in 2019 [2]. 

In an attempt to mitigate the impact of antimicrobial resistance on public health, in 2015, the World Health Organization (WHO) published a Global Action Plan on Antimicrobial Resistance [3], which has been translated to National Action Plans by the member states. The WHO Global Action Plan [3] and other global strategies, such as the political declaration from the 2016 high-level meeting on antimicrobial resistance at the United Nations General Assembly [4] and the FAO/OIE/WHO Tripartite Commitment [5], highlight the importance of the integrated One Health approach to address the antimicrobial resistance challenge. The One Health concept provides a system-level framework to address antibiotic resistance across human, animal, and environmental health [6,7]. Currently, the One Health-based plans focus on the use of antimicrobials and the spread of resistance in human and food animal health sectors, leaving the environmental sector less integrated, and the need to address the environmental sector more comprehensively has been emphasized [7,8,9]. Indeed, in its One Health action plan of 2017, the European Commission specifically addressed environment-related issues of antimicrobial resistance and highlighted the need for monitoring and research [10]. 

The environmental presence of antibiotic resistance-carrying bacteria (ARB) and antibiotic resistance genes (ARGs) [8,9] is recognized as a consequence of past and current antibiotic use [8]. Antibiotics, which are widely applied in human and veterinary medicine and agriculture, can leak into the environment and cycle between the human clinical environment, agricultural and aquacultural settings, pharmaceutical production, water, and land environments. 

Human medicine sectors, particularly hospitals, use a large amount and a broad range of antibiotics in therapeutic measures and present a spot for de novo development of antibiotic resistance as well as for a potential spread of existing ARB between the patients [8]. Hospital effluent wastewater contains an abundance and variety of ARB and ARGs, which can enter the environment [11]. In addition, a large proportion of antibiotics taken by people is excreted into wastewater and, along with sewage from hospitals, enter wastewater treatment plants (WWTP). The antibiotics can be biodegraded, absorbed into sewage sludge, or exit unchanged to the WWTP effluent [12]. The presence of antibiotic resistance bacteria, ARGs, and antibiotics in sub-minimal inhibitory concentrations, which can induce horizontal gene transfer [13], provides a favorable environment for the development, recombination, and spread of antibiotic resistance. Therefore, wastewater treatment plants are often referred to as hotspots for the presence of antibiotic resistance [8].

Likewise, preventive or therapeutic application of antibiotics in livestock and veterinary sectors encourages de novo evolution of antibiotic resistance in animals and contamination of animal excretions with antibiotics in their active form, ARGs, and ARB [14]. Agricultural wastewater, process water from animal husbandry and slaughter facilities, and the spread of manure on fields have been shown to convey antibiotic resistance to the environment [12,15,16]. Soil and aquatic bodies are mostly affected by antibiotic resistance pollution [12,15] and have been recognized as antibiotic resistance reservoirs and sources of further spread [16,17]. In this regard, the WHO and the Advisory Group on Integrated Surveillance of Antimicrobial Resistance (AGISAR), in its standard protocol for integrated multisectoral global surveillance, the “Tricycle protocol”, suggest community and animal slaughter wastewater and surface river waters as suitable sites for monitoring antibiotic resistance in the environment [18]. 

Additionally, the literature indicates wild animals as relevant for disseminating antibiotic resistance in the environment as well as a suitable indicator. Wild animals can encounter contaminated water and food sources, acquire antibiotic resistance, and further contribute to its distribution [19,20]. Notably, migratory birds are recognized as potential disseminators of antibiotic resistance due to their movement over long distances and interaction with diverse ecosystems [21]. These reservoirs of the environment present different sites for quantifying and tracking the spread of antibiotic resistance and changes in its presence over time. 

Overall, as the role of the environment in the spread of antibiotic resistance is becoming more prominent, understanding the involvement of various environmental reservoirs and interconnections between them is of increasing importance to successful surveillance and inhibition of its advancement. In this work, we summarize published research on the occurrence of antibiotic resistance in the environmental reservoirs in three European countries: Austria, Germany, and Switzerland in the form of a narrative review. The countries were selected due to their societal and industrial similarities. For details of the review process please see the Supplemental Files.

## 2. Research Activities on Antibiotic Resistance in Environmental Reservoirs

The occurrence of antibiotic resistance has been studied in various environmental reservoirs. Most evidence was available for aquatic environments, including surface water (*n* = 13) [22,23,24,25,26,27,28,29,30,31,32,33,34] and wastewater sources (*n* = 11) [35,36,37,38,39,40,41,42,43,44,45]. 

**Figure 1:**
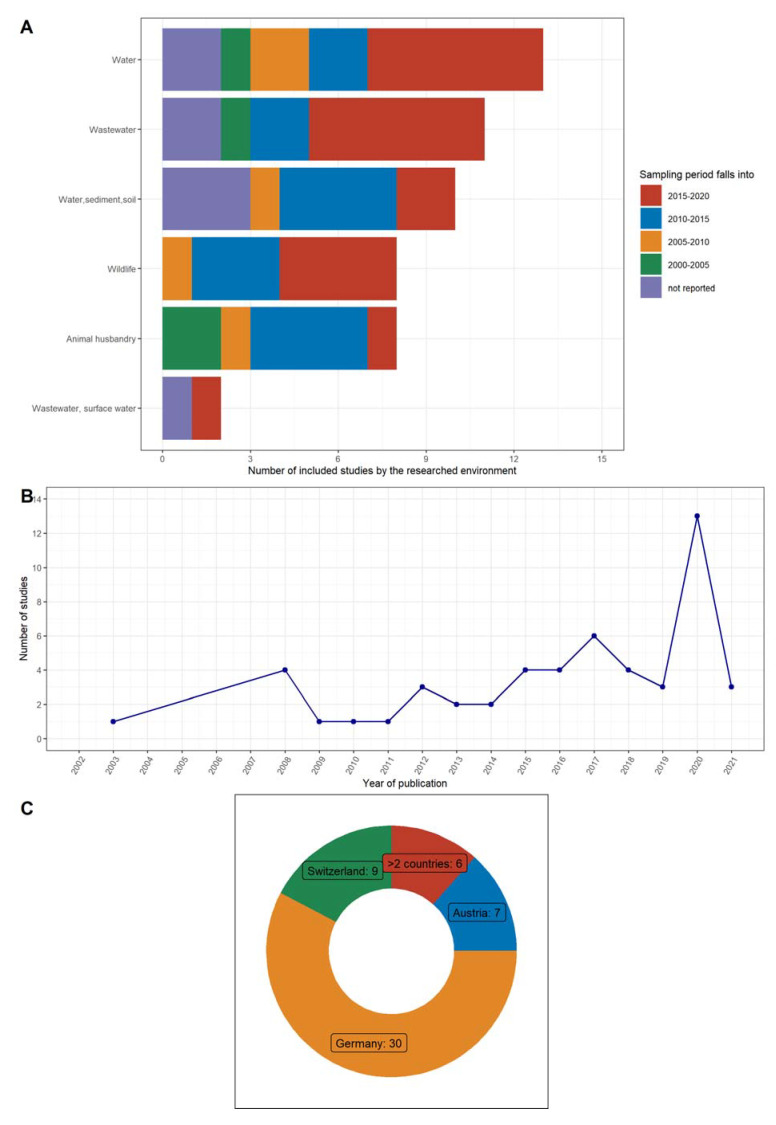
Overview of the studies included (*n* = 52). (**A**) describes the reported sampling periods summarized into 5-year-increments by the source of environment. (**B**) gives the number of studies by publication year. (**C**) presents the number of the included studies by country.

Fewer studies investigated the presence of antibiotic resistance in animal husbandry environments [46,47,48,49,50,51,52,53], wild animals, and birds [54,55,56,57,58,59,60,61]. Less evidence is available for sediment [62,63,64,65,66,67,68] and soil [69,70,71]. Figure 1 and Table S2 give an overview of this literature. The majority of studies (*n* = 20) reported a sampling period between 2015 and 2020. Figure 1A represents the sampling periods summarized into 5-year-increments by the environment source. Figure 1B illustrates the number of studies by publication year and shows that most studies were published in 2020. Both panels suggest an increased research activity in recent years compared to pre-2015. Most of the studies (*n* = 30) in this review, were conducted in Germany [23,24,27,28,29,30,32,36,38,41,42,44,45,46,47,48,49,50,51,52,53,54,57,58,59,60,62,67,72,73], following by Switzerland [24,28,35,41,63,65,66,68,70] and Austria [27,36,42,45,58,71,73] (Figure 1C). Several studies [33,37,39,57,67,72] examined environmental samples from two or more countries, including those selected for this review. Section S2.2 and Figure S2 in Supplementary Materials describe the number of studies conducted in each country stratified by the environmental source. 

For the purposes of this review, phenotypic and genotypic resistance was used without any qualification and quantification of its presence. In case a study reported antibiotic resistance genes, they were translated to the targeted antibiotic classes associated with the genes using Table S1 in Supplementary Materials. The occurrence of antibiotic resistance in the environmental sectors was summarized using the ranking of the WHO list of critically important antimicrobials for human medicine (WHO CIA), the 6th edition (2018): critically important highest priority, critically important high priority, highly important, and important [74]. For that, the antibiotic compounds or compound classes associated with ARGs reported in the studies were identified in the WHO CIA list and assigned the corresponding category. Additionally, resistance to antiseptics, beta-lactams, and ESBL reported in the studies were also included to provide an overview of the findings. 

**Figure 2:**
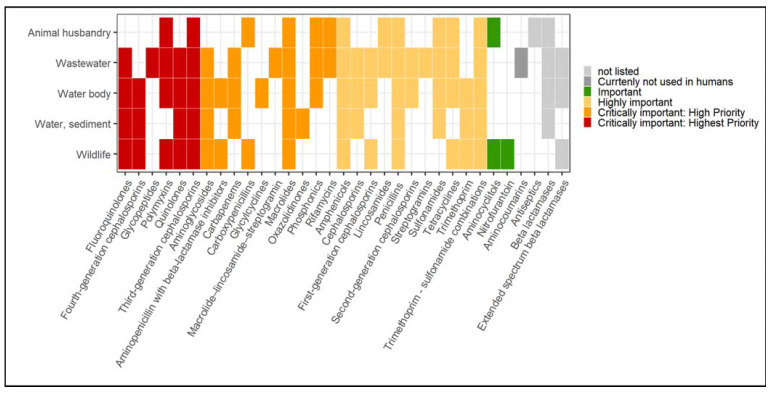
Overview of the occurrence of resistance to antibiotic classes reported in the included studies across the environmental sectors. The “Water, sediment” category also includes soil. The color profile represents the WHO CIA list categories. Notes: ARGs reported in the studies were not assumed to cause the phenotypic antibiotic resistance but rather the resistance potentially associated with the ARGs. Table S8 in Supplementary Materials presents the data.

Potential resistance to one or several antibiotic groups ranked in important to critically important categories could be identified in all environmental sources. Overall, possible resistance to 30 classes indicated in the WHO CIA list could be determined in the included studies (Figure 2). The following sections look at each environment sector separately. In these sections, if a specific antibiotic was not given, the reported antibiotic classes were used.

## 3. Antibiotic Resistance in Water Sources

Water samples from rivers, lakes, bathing areas, coastal waters, ground, and drinking water were investigated [22,23,24,25,26,27,28,29,30,31,32,33,34] (Section S2.3.1 and Table S3 in Supplementary Materials) briefly describe the sampling campaigns. Periodicity and execution of sampling campaigns differed substantially between the studies, including single and iterative sampling. Volumes of collected samples varied from 10 mL to 1.5 L. The reported number of samples ranged from 4 to 164. Out of 13 studies, the number of obtained isolates was reported in four studies [28,29,34,36]. Most of the studies isolated *E. coli* and coliform bacteria.

**Figure 3:**
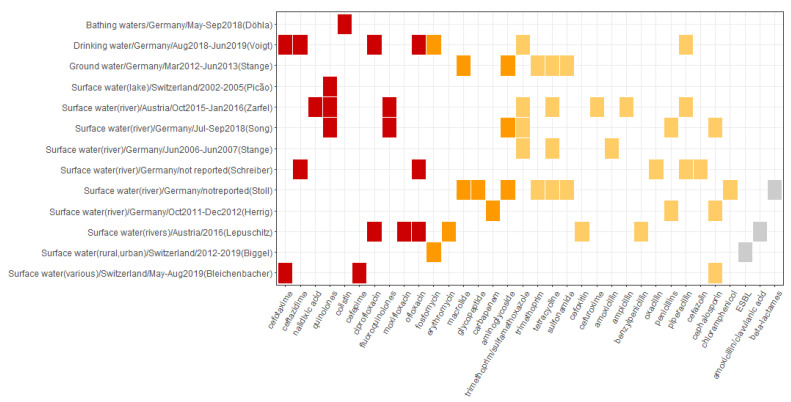
Overview of the occurrence of reported antibiotic resistance patterns in water sources. The horizontal axis depicts resistance patterns as reported in the primary studies and includes antibiotic compounds, antibiotic classes, resistance mechanisms, and ARGs. The vertical axis represents the source of resistance associated with resistance patterns depicted on the horizontal axis as follows: source of sample/Country of study/Sampling period/(First author of the reporting study). The color profile represents the WHO CIA list categories: red, Critically important: Highest Priority; orange, Critically important: High Priority; yellow, Highly important; grey, not listed.

Resistance to antibiotics listed as either critically important or highly important was determined in each study investigating the aquatic environment. Resistance to critically important high and highest priority antibiotics was identified in the majority of the studies (12 out of 13) (Figure 3 and Table S3 in the Supplementary Materials).

Resistance to critically important of highest priority antibiotics was determined in the waters of the Swist river (fluoroquinolone ceftazidime and third-generation cephalosporin ofloxacin) [27] and Weser (classes fluoroquinolones and quinolones) [28], both Germany, as well as in the rivers Danube, Glan, Inn, Traun (fluoroquinolones ciprofloxacin, moxifloxacin, ofloxacin, and aminopenicillin with beta-lactamase inhibitors amoxicillin/clavulanic acid) [25], and Mur (classes fluoroquinolones and quinolones) [34], all in Austria. In Germany, in drinking waters, antibiotic resistance was identified against critically important cefotaxime, ceftazidime, ciprofloxacin, and ofloxacin. The first two are third-generation cephalosporins, and the latter two are fluoroquinolones.

In bathing waters, resistance against colistin (class polymyxins) was detected [23]. The surface waters of lakes and inland streams in Switzerland also tested positive for antibiotic resistance against cefepime (fourth-generation cephalosporins), cefotaxime (third-generation cephalosporins) [22], and quinolones [26]. 

Resistance to critically important of high priority antibiotics was also detected in the Glan, Inn, Traun (macrolide erythromycin) [25], Danube (glycopeptide, macrolide [25,31] and aminoglycoside [31]), Rhine (glycopeptide, macrolides, and aminoglycosides [31]), Weser (class aminoglycosides) [28], and Lahn (class carbapenems) rivers [24]. In drinking water, resistance against fosfomycin [32] (class phosphonics) was found, and in groundwater, resistance against aminoglycosides and macrolides was detected [30]. 

Resistance to nine classes of highly important antibiotics was identified in the water samples. In the river waters, resistance against the following antibiotic classes was reported: first-generation cephalosporins (rivers Swist [27]), second-generation cephalosporins (rivers Danube, Glan, Inn, Traun [25], Mur [34]), cephalosporins (rivers Lanh [24], Weser [28]), penicillins (rivers Swist [27], Rhine [29], Danube, Glan, Inn, Traun [25], Weser [28], Mur [34]), tetracyclines (rivers Rhine [29], Rhine and Danube [31], Mur [34]), trimethoprim-sulfonamide combinations (rivers Rhine [29], Weser [28], Mur [34]), amphenicols (rivers Rhine and Danube [31]), sulfonamides (rivers Rhine and Danube [31]), and trimethoprim (rivers Rhine and Danube [31]).

In the drinking water in Germany, resistance against antibiotic classes penicillins and trimethoprim–sulfonamide combinations was identified in one study [32]. In the groundwater in Germany, resistance against three highly important classes was reported: sulfonamides, tetracyclines, and trimethoprim [30]. In Switzerland, in the surface waters of lakes and streams, resistance to cephalosporins was reported in one study [22]. Resistance to the antibiotics listed as important was not reported for the aquatic environments. Additionally, the presence of ARGs coding beta-lactamases in rivers Rhine and Danube [31] were found. 

## 4. Antibiotic Resistance in Wastewater

Wastewater, sewage, and sludge collected in different wastewater treatment plants were a frequent subject of research [35,36,37,38,39,40,41,42,43,44,45]. Aquatic environments, which receive wastewater discharges, were also studied [72,73]. Collectively, the current studies of wastewater provide more comprehensive and diverse analyses compared to other reservoirs. The research objectives of the studies varied; therefore, so did the study designs and methods. Of these 13 studies, six looked at the antibiotic resistance in wastewater in a more general context [35,36,37,38,72,73]. Mainly, influents and effluents of wastewater treatment plants were investigated. Seven other studies [39,40,41,42,43,44,45] conducted more targeted analyses (details are given in Section S2.3.2 in Supplementary Materials).

**Figure 4:**
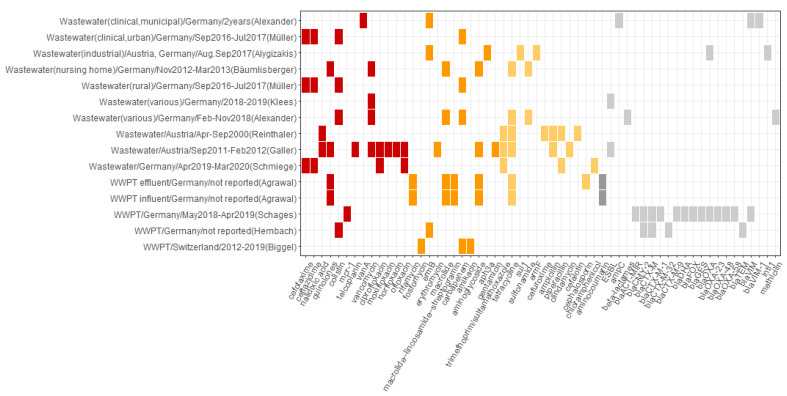
Overview of the occurrence of reported antibiotic resistance patterns in wastewater. The horizontal axis depicts resistance patterns as reported in the primary studies and includes antibiotic compounds, antibiotic classes, resistance mechanisms, and ARGs. The vertical axis represents the source of resistance associated with resistance patterns depicted on the horizontal axis as follows: source of sample/Country of study/Sampling period/(First author of the reporting study). The color profile represents the WHO CIA list categories: red, Critically important: Highest Priority; orange, Critically important: High Priority; yellow, Highly important; grey, not listed.

The sampling strategies varied greatly regarding sampling methods, sites, number of samples at each site, and periodicity. Types of samples included grab samples, 24-h-composite samples, and wastewater collected over one year. The sampling periodicity included a single sampling campaign, five sampling days, and periodical sampling each week, once per month, twice a month, or every six weeks. The number of obtained samples varied correspondingly from 4 to 128. Details of the sources, sampling, and targets are summarized in Table S4 in Supplementary Materials. Mostly, the studies looked at the occurrence and distribution of antimicrobial-resistant *Enterobacterales* and various antibiotic resistance genes in waters different in their quality and proximity to WWTPs. All studies reported the occurrence of potential resistance to at least one or more antibiotic classes in the three WHO CIA list categories: critically important of highest and high priority and highly important (Figure 4 and Table S4 in Supplementary Materials).

Resistance to antibiotic compounds listed as critically important of highest priority was reported in most studies. Resistance to fluoroquinolones was indicated in activated sludge in the area of Graz in Austria (to ciprofloxacin, moxifloxacin, norfloxacin, and ofloxacin [40]) and in wastewater collected in socio-spatially different districts in Germany (ciprofloxacin and ofloxacin [45]). Similarly, resistance to glycopeptides was reported in the studies from Germany and Austria. Teicoplanin resistance was found in activated sludge in Graz [40]. Resistance to vancomycin was more investigated and identified in five studies that looked at wastewater from 23 conventionally-treated WWTPs [36] and wastewater in Germany [40,74,75], and activated sludge in Austria [40]. Resistance to colistin in wastewater was reported in three German studies [38,43,44], and the presence of the *mcr-1* gene, which is associated with resistance to colistin, was identified in one study [44]. Resistance to quinolones was identified in four studies: two from Germany [35,38] and two from Austria [40,43]. Furthermore, two German studies identified resistance to third-generation cephalosporins (cefotaxime and ceftazidime) in clinical, urban, and rural wastewater [42,45].

Patterns of resistance to antibiotics listed in six classes ranked as critically important of high priority in WHO CIA were identified. The six classes were aminoglycosides, carbapenems, macrolide–lincosamide–streptogramin (MLS), macrolides, phosphonics, and rifamycins. Resistance to macrolides was most frequently reported [35,36,37,38,40,41]. For aminoglycosides, most studies did not specify antibiotic substances. The resistance was reported in two German [35,38] and one Austrian [40] study of wastewater. Additionally, resistance to amikacin was found in wastewater in Switzerland [39]. Resistance to rifamycins was detected in one German study [35] and to phosphonics (fosfomycin) in one study from Switzerland [39]. 

Furthermore, the studies that investigated wastewater reported either phenotypic or genotypic resistance against compounds named in nine classes listed as highly important in the WHO CIA list: amphenicols, first- and second-generation cephalosporins, lincosamides, penicillins, streptogramins, sulfonamides, tetracyclines, and trimethoprim–sulfonamide combinations. The most frequently reported resistance was tetracyclines [37,38,40,42,45] and sulfonamides [36,37,38]. 

The presence of ARGs coding beta-lactamases was reported in five studies [38,39,43,46,74] and organisms harboring extended-spectrum beta-lactamases in two studies [40,73]. 

## 5. Antibiotic Resistance in the Environment of Animal Husbandry

The studies examining the spread of antibiotic resistance from animal husbandry to the environment analyzed mostly manure and wastewater [46,47,48,49,50,51,52,53]. All studies looked at samples collected in livestock farms in Germany. The criteria, which would allow a clear definition of an environment-related sample, were not well established for this environmental reservoir. Two [50,53] investigated antibiotic resistance in farms in a more general context among these eight studies. Other six studies conducted more targeted analyses (see Section S2.3.3 in Supplementary Materials).

**Figure 5:**
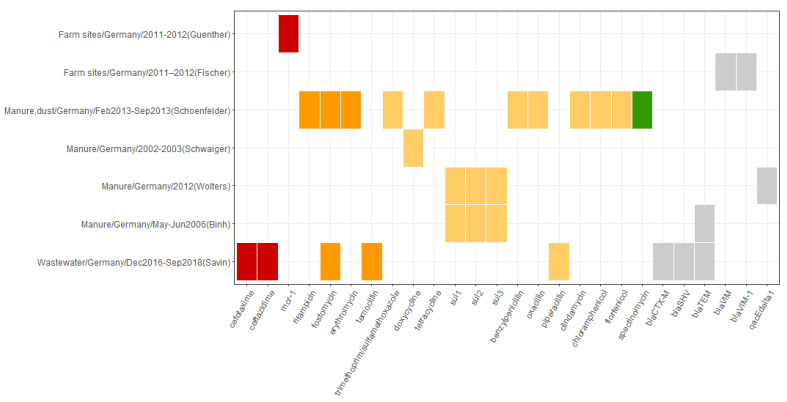
Overview of the occurrence of reported antibiotic resistance patterns in the environment of animal husbandry. The horizontal axis depicts resistance patterns as reported in the primary studies and includes antibiotic compounds, antibiotic classes, resistance mechanisms, and ARGs. The vertical axis represents the source of resistance associated with resistance patterns depicted on the horizontal axis as follows: source of sample/Country of study/Sampling period/(First author of the reporting study). The color profile represents the WHO CIA list categories: red, Critically important: Highest Priority; orange, Critically important: High Priority; yellow, Highly important; green, Important; grey, not listed.

The studies varied in the origin of the samples and sampling strategies. The number of samples varied between 3 and 380. Figure 5 illustrates the occurrence of antibiotic resistance in the environment of animal husbandry, which could be extracted from the included studies. Table S5 presents the details on the sampling and the reported antibiotic resistance patterns.

Only two studies reported the WHO CIA’s critical importance of the highest priority substances [48,50]. Savin et al. [50] found *E. coli* resistant to cefotaxime and ceftazidime (third-generation cephalosporins) in process water and wastewater samples collected at different sites and production stages in German poultry slaughterhouses. Guenther et al. [48] reported the presence of *E. coli* positive for *mcr-1* ARG, which is associated with resistance to colistin, in the samples obtained from boot swabs, dog feces, stable flies, and manure in pig farms. 

Among WHO CIA’s critically important of high priority classes, resistance to carboxypenicillins, macrolides, phosphonics, and rifamycins was identified in the studies. In the dust and manure samples in the study by Schoenfelder et al. [51], resistance to erythromycin (macrolides), rifampicin (rifamycins), and fosfomycin (phosphonics) was identified. In the processed water in poultry slaughterhouses, resistance to temocillin (carboxypenicillins) and fosfomycin was reported [50].

More studies reported resistance to highly important substances. The antibiotic classes included amphenicols, lincosamides, penicillins, sulfonamides, tetracyclines, and trimethoprim–sulfonamide combinations. Resistance to most of them was identified in the study by Schoenfelder et al. [51]. The authors reported resistance to chloramphenicol, florfenicol, clindamycin, benzylpenicillin, oxacillin, tetracycline, and trimethoprim/sulfamethoxazole detected in dust and liquid manure samples from pig farms. Additionally, the study identified resistance to spectinomycin, which belongs to aminocyclitols listed as important in WHO CIA. ARGs associated with resistance to sulfonamides (*sul*1, *sul*2, *sul*3) were identified in the samples of pig manure in two studies [46,53]. Resistance to doxycycline (tetracyclines) was found in pig manure in one study [52]. The presence of beta-lactamases ARGs (Table S5 in Supplementary Materials) was reported in different samples originating from farms: piggery manure [46], wastewater [50], and emission source of a farm (single feces, manure, flies) [47]. 

## 6. Antibiotic Resistance in Wildlife

Eight studies [54,55,56,57,58,59,60,61] in this review examined the occurrence of antibiotic resistance in wildlife. Of them, three studies investigated wild birds [58,61,63], and one each, mouflons [55], wild boars [58], wild rodents [54], and flies [60]. One study [57] examined four types of animals: wild boars, roe deer, wild ducks and geese. Concerning the screening for antibiotic resistance, only one study explored the presence of a range of bacteria in wildlife [57]. The other seven studies looked at specific bacterial species or resistance patterns. Section S2.3.4 in Supplementary Materials overviews the details. Overall, the studies, which investigated antibiotic resistance in wildlife, differed in the studied animals, geographical sites, type of samples, duration of the sampling, and the targets for screening. The number of samples varied from 32 to 1443. Table S6 summarizes the sampling and reported occurrence of antibiotic resistance in wildlife. 

**Figure 6:**
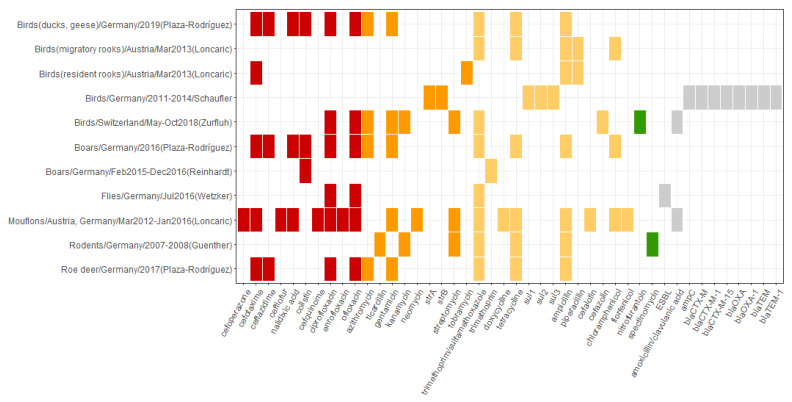
Overview of the occurrence of reported antibiotic resistance patterns in wildlife. The horizontal axis depicts resistance patterns as reported in the primary studies and includes antibiotic compounds, antibiotic classes, resistance mechanisms, and ARGs. The vertical axis represents the source of resistance associated with resistance patterns depicted on the horizontal axis as follows: source of sample/Country of study/Sampling period/(First author of the reporting study). The color profile represents the WHO CIA list categories: red, Critically important: Highest Priority; orange, Critically important: High Priority; yellow, Highly important; green, Important; grey, not listed.

Resistance to different compounds listed in all categories of WHO CIA was identified in wild animals (Figure 6). Resistance to compounds listed in critically important of highest priority group was identified in all studied animals except rodents and migratory rooks. The majority of compounds were identified in the studies, which examined wild boars, mouflons, ducks and geese. Two German studies which analyzed samples from boars reported different results. In the samples originating from hunted wild boars, Plaza-Rodríguez et al. [57] reported resistance to ciprofloxacin, ofloxacin, colistin, nalidixic acid, cefotaxime, and ceftazidime. Reinhardt et al. [58] identified resistance to colistin, although the authors looked only at *Yersinia pseudotuberculosis* compared to the more general approach by Plaza-Rodríguez et al. Moreover, Plaza-Rodríguez et al. [57] reported resistance to the same antibiotics found in wild ducks and geese. Similarly, in mouflons, resistance to ciprofloxacin, enrofloxacin, ofloxacin, cefquinome, cefoperazone, cefotaxime, and ceftiofur was identified [55]. In roe deer, resistance to fewer compounds in this category was found: ciprofloxacin, ofloxacin, cefotaxime, and ceftazidime [57]. Resistance to only two critically important of highest priority compounds was found in flies originating from the urban center in Berlin, ciprofloxacin, and ofloxacin (fluoroquinolones) [60], and none in wild rodents [54]. In wild birds, resistance to three antibiotics of highest priority was reported: ciprofloxacin and ofloxacin in wild birds in Switzerland [61], and cefotaxime in resident rooks in Austria [56]. Additionally, resistance to multiple compounds listed as critically important of high priority and highly important in WHO CIA was identified in the included studies. The highest number of compounds was reported in mouflons in Austria and Germany [55]. Aminoglycosides were the most frequently reported class. Resistance to gentamicin was identified in mouflons [55], wild boars [57], wild ducks and geese [57], roe deer [57], and wild birds in Switzerland [61]. Resistance to streptomycin was reported in wild rodents [54], mouflons [55], and birds [61]. Additionally, in the German study of wild birds, the presence of ARGs *str*A/B, which are associated with streptomycin, was indicated [59]. Furthermore, resistance to kanamycin (in rodents and wild birds), neomycin (mouflons), and tobramycin (resident rooks) was found. Other classes of high priority include aminopenicillins with beta-lactamase inhibitors (amoxicillin/clavulanic acid in mouflons and birds), carboxypenicillins (ticarcillin in rodents), macrolides (azithromycin in boars, roe deer, ducks and geese, and birds).

Resistance to highly important antibiotic compounds was found in all examined wildlife species. The antibiotic classes included amphenicols, first-generation cephalosporins, penicillins, sulfonamides, tetracyclines, trimethoprim, and trimethoprim–sulfonamide combinations. The most frequently reported resistance was against ampicillin, tetracycline, and trimethoprim/sulfamethoxazole. The first two were associated with rodents, mouflons, migratory rooks, resident rooks, boars, roe deer, ducks and geese. The resistance to the latter was also seen in flies and wild birds. In addition, among penicillins, resistance to piperacillin was reported in migratory and resident rooks in Austria [56]. Resistance to first-generation cephalosporins was present in mouflons (against cefalotin) and birds in Switzerland (against cefazolin) [61]. Among the amphenicols, resistance to chloramphenicol was reported in mouflons [55], migratory rooks [56], and boars [57], and to florfenicol in mouflons [55]. Further, resistance to trimethoprim was reported in wild boars [58] and to doxycycline in mouflons [55]. The presence of antibiotic-resistance genes *sul*1, *sul*2, *sul*3 associated with resistance to sulfonamides was identified in the samples collected in wild birds in Germany [59]. 

Besides the critically and highly important classes of compounds, the occurrence of resistance to two antibiotics listed as important was reported. These are resistance to spectinomycin in wild rodents in Germany [54] and nitrofurantoin in wild birds in Switzerland [61]. Additionally, the presence of ARGs associated with resistance to beta-lactams was investigated and reported in the study of wild birds in Germany [59]: *bla*_CTX-M_, *bla*_CTX-M-1_, *bla*_CTX-M-15_, *bla*_OXA_, *bla*_OXA-1_, *bla*_TEM_, *bla*_TEM_*-*_1_. Table S6 in Supplementary Materials gives more details on the reported presence and the resistance profiles identified in the studies.

## 7. Antibiotic Resistance in Sediment and Soil

Less evidence is available for sediment and soil samples compared to other environmental sectors [62,63,64,65,66,67,68,69,70,71]. Five out of seven studies that looked at sediment were conducted in Switzerland [63,64,65,66,68] and two in Germany [62,67]. Mostly, the samples of sediment were collected together with water samples. Two studies investigated only soil samples, one in Germany [70] and one in Austria [71]. One study examined water and soil in Austria [69].

Out of five Swiss studies, four conducted sediment sampling in the Bay of Vidy and lake Geneva [63,64,65,68] and one in Lake Brêt [66]. One of the German studies examined the river sediment (the Kraichbach River, Baden-Württemberg) [67] and another the coastline sediment of Baltic and North seas [62]. One study investigated soil obtained from Berlin Tiergarten, an urban park, and an abandoned sewage field in Berlin [70]. One Austrian study sampled the soil from 50 maize fields and 50 potato fields [71]. Another studied the occurrence of *Listeria monocytogenes* in the samples of water and soils with different compositions at different altitudes [69]. 

Only one study [66] in this group conducted the sampling later than 2015. Three studies [65,70,72] did not report the sampling period. The studies used various sites and depth or extraction layers for sampling. The number of samples varied from 6 to 467, although five (all from Switzerland) [63,64,65,66,68] out of ten studies did not report the number of obtained samples. Table S7 in Supplementary Materials details the locality, sites, and sampling strategies reported in the studies. 

**Figure 7:**
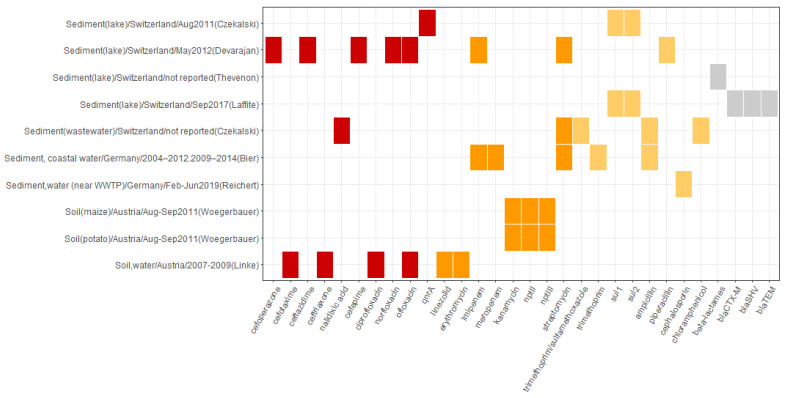
Overview of the occurrence of reported antibiotic resistance patterns in sediment and soil. The horizontal axis depicts resistance patterns as reported in the primary studies and includes antibiotic compounds, antibiotic classes, resistance mechanisms, and ARGs. The vertical axis represents the source of resistance associated with resistance patterns depicted on the horizontal axis as follows: source of sample/Country of study/Sampling period/(First author of the reporting study). The color profile represents the WHO CIA list categories: red, Critically important: Highest Priority; orange, Critically important: High Priority; yellow, Highly important; grey, not listed.

Compared to other environmental reservoirs, resistance to compounds in the critically important of highest priority group were less frequently reported for sediment and soil (Figure 7 and Table S7 in Supplementary Materials). Most of them were identified in the studies of sediment by Devarajan et al. [65] and soil by Linke et al. [69], although the compounds differed. The Swiss studies reported the presence of resistance to norfloxacin, ofloxacin, cefepime, nalidixic acid, and ceftazidime in the sediment samples. In sediments of Lake Geneva, the occurrence of the *qnr*A gene associated with resistance to fluoroquinolones was reported [64]. Resistance to ciprofloxacin, ofloxacin, cefotaxime, and ceftriaxone was identified in the Austrian soil, sampled at different altitudes [69].

More studies reported the occurrence of resistance to compounds listed as critically important of high priority, or highly important. No compounds of category “important” were named. In the sediment samples, resistance to streptomycin was reported in three studies (two Swiss [64,65] and one German [62]) and to imipenem in two studies [62,65]. For soil, the presence of *nptII* and *nptIII* genes were found in both potato and maize fields in Austria [71], as well as resistance to linezolid and erythromycin in another Austrian study [69]. No ARGs were identified in the soil samples in Germany [70].

Regarding the highly important WHO CIA category, resistance to chloramphenicol, ampicillin, piperacillin, trimethoprim, and trimethoprim/sulfamethoxazole was reported for sediment samples. Additionally, the presence of sulfonamide resistance genes in sediments of both freshwater lakes in Switzerland, Lake Geneva [64] and Lake Brêt [66], was reported. The presence of resistance to beta-lactams was identified in two studies analyzing Lake Geneva sediment [66,68]. 

## 8. Discussion

This review describes the current landscape of epidemiological research on the occurrence of antibiotic resistance in the environment in Austria, Germany, and Switzerland. A precise classification of the studies by environmental sectors was not feasible due to the differences in the sampling sources. Many studies explored multiple sources of the environment following their specific research objectives. Even after considering them by the environment sectors, the studies differed considerably in their research foci, designs, targeted bacteria, sampling periods, sites and strategies, and the reporting of the results. The studies on aquatic environments often examined wastewater and surface water of water bodies or sediments. The studies examining antibiotic resistance in animal husbandry analyzed manure samples most frequently and included farm samples, such as dust and boot swabs. The studies investigating wildlife showed more considerable heterogeneity in their designs and studied animals. This review did not identify two studies that examined comparable locality and wild animals. 

Wastewater, sewage, and sludge collected in different wastewater treatment plants were the most investigated environmental reservoirs. Given that WWTPs are recognized as hotspots for developing and disseminating antibiotic resistance, systematic investigation of antibiotic resistance in WWTP is not surprising. As receiving freshwater serves as an essential part of drinking water for the public, monitoring the dispersion of antibiotic resistance in the aquatic environment is of public health importance. One study in this review conducted extensive research on the monitoring strategies for the spread of antibiotic resistance in rivers [67] and concluded that water samples could reflect temporal and seasonal changes in the occurrence of antibiotic resistance. However, the authors also recommended increasing the usage of sediment and biofilm samples in the monitoring strategies of aquatic ecosystems. Indeed, the Swiss studies that looked at antibiotic resistance in freshwaters frequently analyzed sediment samples along with water grabs. Monitoring of bathing sites was also of research interest, however, less represented in this review. Although the transmission of antibiotic-resistant bacteria and ARGs from drinking or recreational waters to humans is suggested to be possible, none of the included studies provided an epidemiological investigation that would link the dispersion of antibiotic resistance in the aquatic environment to the prevalence of it in the human population. 

Analogously, the studies that investigated the presence of antibiotic resistance in manure did not further examine its spread as a fertilizer on fields and the presence of antibiotic resistance in the receiving soils. Resistant bacteria from agricultural soils could spread to drinking water and vegetables [75]; therefore, surveying this pathway is essential for capturing the environmental role in spreading antibiotic resistance. The evidence base of resistance in soils is poor in the considered countries. The review identified only three studies that examined soil samples. 

Investigation of the occurrence of ARBs and ARGs in wild animals can serve as an indicator of the spread of the resistance through environmental pathways [19]. Although it is not suggested in the Tricycle protocol, screening wild animals can contribute to grasping the environmental dispersion of antibiotic resistance. The studies in this review, which focused on the wildlife, mainly explored animals that people may consume as game meat, e.g., wild boars. Fewer studies investigated other animals and birds, which could serve as bioindicators of the spread of antibiotic resistance in the environment. The diversity of the approaches to studying antibiotic resistance in wildlife suggests a need for methodological research on conducting surveys of wildlife that aim to be informative about the pathways of spread and acquisition of antibiotic resistance in the environment. For this, ecological context along with epidemiological considerations is required. Ramey et al. suggest that the studies incorporate ecologic factors, such as “habitat use, foraging strategy, and anthropogenic inputs into the environment” [19], into the surveys. 

The occurrence of antibiotic resistance in the environment was summarized using the categories of the WHO CIA list. The review showed that antibiotic resistance to critically important and highly important substances was identified in all environmental reservoirs. Moreover, the resistance to antibiotics in these categories was most frequently reported as compared to the “important” category. However, it cannot be concluded whether the more frequent observation of resistance to critically important and highly important antibiotics was due to the sampling strategies and targeted antibiotic susceptibility testing or the actual state of the environmental dissemination of resistance to these substances. A more systematic analysis of the methods and isolation protocols is required to interpret the findings. 

The approaches to quantify the presence of antibiotic resistance differed considerably between the studies. It suggests that the current research landscape presents rather data-gathering efforts than interpretative studies or systematic surveillance. Comparing the occurrence and abundance of antibiotic resistance between the environmental reservoirs requires methodological consistency in quantifying environmental dispersion. However, developing a unified protocol for extensive analyses and large-scale sampling for antibiotic-resistant bacteria and/or antibiotic-resistance genes in environmental reservoirs poses considerable challenges. These include, but are not limited to, considerations of (i) representativeness of selected sampling sites and sample size given the variety and expanse of environmental reservoirs, (ii) detection techniques for both phenotypic and genotypic resistance (culture-based techniques, PCR, qPCR, or metagenomic analysis), (iii) methods for quantification of prevalence, (iv) influence of external factors on the accuracy of accompanying epidemiological data, such as antibiotic contamination, climate, and other drivers of resistance in the area. Although the Tricycle protocol provides detailed guidelines for surveillance of ESBL-producing *E. coli* in the One Health sectors, further protocols should be devised for other indicators of antibiotic resistance specific to an environmental reservoir. Furthermore, this review did not identify studies that simultaneously provided an epidemiological investigation in all three One Health sectors, as suggested in the Tricycle protocol. The search strategy might not be sensitive enough to identify such studies in the current literature base. However, it is more likely that studies investigating antibiotic resistance at the human–animal–environment interface in the considered countries are rare or absent. 

This review has certain limitations. Methods of isolation and identification of antibiotic resistance were not extracted and analyzed in this review. They are likely to contribute additionally to methodological heterogeneity between the studies. The differences between the studies made comparison and synthesis of available evidence challenging. Therefore, the quantified presence was extracted and reported but not synthesized in this review. Moreover, the reporting of antibiotic resistance occurrence was not stratified by bacteria species, which would provide a more detailed review. Despite these limitations, this study offers an overview of German, Austrian, and Swiss studies on the general occurrence of phenotypic and genotypic resistance in different environmental reservoirs. 

An update of this review is needed to include methodological aspects (such as isolate collection, sites, susceptibility testing, and quality control [76]) of the selected studies and to provide a meta-analysis of the reported outcome of antibiotic resistance across the environmental reservoirs. Assessment of methodological characteristics of the study designs would be beneficial and informative for interpretation and evidence synthesis. However, quality evaluation within a review framework would require a quality assessment instrument that would allow the appraisal of epidemiological, environmental, and ecological aspects of the studies. Additionally, more comprehensive studies, which apply a consistent protocol and look at antibiotic and antibiotic resistance pollution across the environmental reservoirs and over time, are needed to interpret the environmental dissemination of antibiotic resistance. Understanding and managing antibiotic resistance transmission and spread require a more holistic approach to investigating antibiotic resistance dissemination and epidemiological research across the sectors of One Health. 

## 9. Conclusions

This work aimed to provide an overview of research on the antibiotic resistance present in the environment conducted in Austria, Germany, and Switzerland. Although the number of studies is increasing and research effort is ongoing, the current state of research resembles a non-harmonized patchwork of data-gathering efforts. The current state of research in Austria, Germany, and Switzerland requires collecting more evidence for antibiotic resistance spread in soil and wildlife and the transmission pathways through the environmental reservoirs. The heterogeneity between the studies poses challenges for evidence synthesis and interpretation of the collective findings. Therefore, the field would benefit from advancing the research efforts on methodological aspects and applying good research practice in surveying antibiotic resistance in the environmental reservoirs. The importance of looking at the environmental sector of One Health cannot be underestimated, and a more holistic approach to the research is required to facilitate interpretation and connect the spread of antibiotic resistance in the environment to the other two levels of One Health.

## Data Availability

The data reported in the present review were obtained from the included articles, which are cited in the main text. The data generated within this review are presented in the tables in the main text and Supplemental Materials.

## Supplementary Materials

The following supporting information can be downloaded at: https://www.mdpi.com/article/10.3390/microorganisms10040728/s1, Figure S1: Results of the searches and study selection represented following the Prisma chart; Figure S2: Number of studies by country and environment; Table S1: Translation of reported antibiotic-resistance gene (ARG) to antibiotic compound or antibiotic class; Table S2: Overview of the included studies (*n* = 52); Table S3: Presence of antibiotic resistance or antibiotic resistance genes in water bodies as reported in the included studies (*n* = 13); Table S4: Presence of antibiotic resistance or antibiotic resistance genes in wastewater as reported in the included studies (*n* = 13); Table S5: Presence of antibiotic resistance or antibiotic resistance genes in the environment of animal husbandry as reported in the included studies (*n* = 8); Table S6: Presence of antibiotic resistance or antibiotic resistance genes in wildlife as reported in the included studies (*n* = 8); Table S7: Presence of antibiotic resistance or antibiotic resistance genes in sediment and soil as reported in the included studies (*n* = 10); Table S8: Reported resistance patterns categorized by associated antibiotic class, WHO CIA list rank and environmental reservoir. Reference [77] is cited in the supplementary materials.

## Abbreviations

AMR	antimicrobial-resistant
ARB	antibiotic resistance-carrying bacteria
ARG	antibiotic resistance gene
CoNS	coagulase-negative staphylococci
CPE	carbapenemase-producing Enterobacteriaceae
DART	the German Antimicrobial Resistance Strategy
ESBL	extended spectrum beta-lactamases
FAO/OIE/WHO	the Food and Agriculture Organization of the UN/the World Organisation for Animal Health/the World Health Organisation
GLASS	the Global Antimicrobial Resistance Surveillance System
MDR	multidrug-resistant
MRGN	multidrug-resistant Gram-negative
MRSA	methicillin-resistant Staphylococcus aureus
MSSA	methicillin-susceptible Staphylococcus aureus
STEC	Shiga toxin-producing Escherichia coli
VRE	Vancomycin-resistant Enterococcus
WHO	the World Health Organisation
WWTP	wastewater treatment plant
XDR	extensively drug-resistant
