# Supplementary material for: Occurrence of Antimicrobial Resistance in the Environment in Germany, Austria, and Switzerland: A Narrative Review of Existing Evidence
Source: Microorganisms. 2022 Mar 29;10(4):728. doi: 10.3390/microorganisms10040728 (PMC9027620; doi:10.3390/microorganisms10040728)
Supplement: Supplementary file 1 [file microorganisms-10-00728-s001.zip › microorganisms-1600618-supplementary.pdf]

Supplement

Occurrence of antimicrobial resistance in the environment in Germany, Austria and Switzerland: a narrative review of existing evidence

Marina Treskova, Alexander Kuhlmann, Fritjof Freise, Lothar Kreienbrock and Sandra Brogden.

Content

1. Methods 2
   1.1. Searches, literature screening, study selection and data extraction 2
      1.1.1. Search syntax for EMBASE 2
      1.1.2. Search syntax for CAB Abstracts 2
   1.2. Study selection criteria 2
   1.3. Data extraction 3
   1.4. Translation of reported antibiotic-resistance gene (ARG) to antibiotic compound or antibiotic class 4
2. Included literature 5
   2.1. Results of the searches and study selection 5
   2.2. Overview of the included studies 6
   2.3. Overview of the sampling strategies 7
      2.3.1. Antibiotic resistance in water sources. 7
      2.3.2. Antibiotic resistance in wastewater 7
      2.3.3. Antibiotic resistance in the environment of animal husbandry 8
      2.3.4. Antibiotic resistance in wildlife 8
      2.3.5. Antibiotic resistance in sediment and soil 9
   2.4. Results tables 11
   2.5. Excluded studies 50

1. Methods

1.1. Searches, literature screening, study selection and data extraction

Two publication search engines, EMBASE (including Medline and Pubmed) and CAB Abstracts were used to identify peer-reviewed epidemiological studies, which explored occurrence of antibiotic resistance in the environment. The search syntaxes were used as described below.

1.1.1. Search syntax for EMBASE

((antibiotic:ti,ab AND resistant:ti,ab) OR (antibiotic:ti,ab AND resistance:ti,ab) OR (anti*:ti,ab AND resist*:ti,ab) OR 'multi-drug-resistant':ti,ab)
AND ('water':ti,ab OR 'waste':ti,ab OR 'sewage':ti,ab OR 'manure':ti,ab OR 'sludge':ti,ab OR 'environment':ti,ab OR 'soil':ti,ab OR soil*:ti,ab OR environment*:ti,ab OR 'land':ti,ab OR plant*:ti,ab OR (human:ti,ab AND environment:ti,ab) OR (animal:ti,ab AND environment:ti,ab) OR (human:ti,ab AND water:ti,ab) OR (human:ti,ab AND soil:ti,ab) OR (hospital:ti,ab AND environment:ti,ab) OR (hospital:ti,ab AND water:ti,ab) OR (hospital:ti,ab AND soil:ti,ab) OR (livestock:ti,ab AND environment:ti,ab) OR (livestock:ti,ab AND soil:ti,ab) OR (livestock:ti,ab AND water:ti,ab) OR (industry:ti,ab AND environment:ti,ab) OR (industry:ti,ab AND soil:ti,ab) OR (industry:ti,ab AND water:ti,ab) OR (aquaculture:ti,ab AND environment:ti,ab) OR (aquaculture:ti,ab AND soil:ti,ab) OR (aquaculture:ti,ab AND water:ti,ab) OR (agriculture:ti,ab AND environment:ti,ab) OR (agriculture:ti,ab AND soil:ti,ab) OR (agriculture:ti,ab AND water:ti,ab)) AND ('germany':ti,ab OR 'switzerland':ti,ab OR 'austria':ti,ab) AND ([english]/lim OR [german]/lim) AND ([embase]/lim OR [medline]/lim OR [pubmed-not-medline]/lim)
AND [2000-2021]/py
ti,ab stands for "title and abstract"

1.1.2. Search syntax for CAB Abstracts

(ab:("antibiotic resistant" OR "antibiotic resistance")
AND ab:(water OR waste OR sewage OR manure OR sludge OR environment OR soil OR land OR plant OR (human AND environment) OR (animal AND environment) OR (human AND water) OR (human AND soil) OR (hospital AND environment) OR (hospital AND water) OR (hospital AND soil) OR (livestock AND environment) OR (livestock AND soil) OR (livestock AND water) OR (industry AND environment) OR (industry AND soil) OR (industry AND water) OR (aquaculture AND environment) OR (aquaculture AND soil) OR (aquaculture AND water) OR (agriculture AND environment) OR (agriculture AND soil) OR (agriculture AND water))
AND ab:(Germany OR Denmark OR France OR Netherlands OR Switzerland OR Austria))
AND (((item-type:(("Journal article")))))

The results of searches were exported into Endnote (X7.7). After the automatic removal of duplicates, titles and abstracts of the identified de-duplicated records were screened for inclusion.

1.2. Study selection criteria

The search was restricted to the epidemiological studies conducted in Austria, Germany, and Switzerland after the year 2000. Quantification of the environmental occurrence of chemical compounds known to be resistance drivers, such as antibiotics, biocides, and heavy metals, was outside the scope of this review.

For inclusion into this review, a paper has to meet the following criteria:

i. Peer-reviewed original study

ii. Investigates the occurrence of antibiotic resistance in the environment
    - Environment includes water bodies, wastewater, surface water, grey or reclaimed water, sewage, soil, land, and wildlife
    - Antibiotic resistance includes phenotypic or genotypic definition of resistance

iii. Epidemiological study in terms of non-experimental study design

iv. Report the results for the samples obtained in the environment

v. Conducted in Germany, Switzerland or Austria or conducted research in multiple countries including Germany, Switzerland or Austria and report the results separately

vi. English or German language

vii. Published during 2000 - 2021

Study was excluded based on the following criteria:

ii. Systematic review or meta-analysis

iii. Experimental study

iv. Investigates azole resistance of environmental and clinical *Aspergillus fumigatus*

v. Does not investigate occurrence of antibiotic resistance

vi. Investigate following reservoirs
    - Hospital environment
    - Food sources
    - Pets and veterinary clinic environment
    - The areas directly surrounding the farm animals or any other facility of food production chain (e.g. dust in slaughterhouse)

vii. No samples from the environment were collected and examined

viii. Focused only on humans, animals or interaction between humans and animals

ix. Conducted outside of Germany, Switzerland and Austria

x. Investigated or quantified the occurrence of resistance-driving chemical compounds including antibiotics, metals, genes and biocides

xi. Published before 2000

xii. Other than a peer-reviewed journal article

xiii. Do not focus on the occurrence of antibiotic resistance

xiv. Conference abstracts

1.3. Data extraction

To facilitate a narrative review, the following data were extracted from the identified studies to a predefined table: authors, year, journal, country, geographical location of samples origin, research focus, examined environment, sampling period, sampling strategy, targeted antibiotic-resistant bacteria

or antibiotic resistance genes, number of samples, number of isolates, reported occurrence of antibiotic resistance. Data were extracted using Microsoft Excel.
MTS conducted the literature search, the study selection, and data extraction followed the established review protocol. The study selection and data extraction were confirmed by the second reviewer (AK). Any disagreements were resolved via discussion with a third reviewer (LK).

1.4. Translation of reported antibiotic-resistance gene (ARG) to antibiotic compound or antibiotic class

Table S1. Translation of reported antibiotic-resistance gene (ARG) to antibiotic compound or antibiotic class

**Reported ARG**	**Associated antibiotic class**	**Reference**
ampC	Ampicillin, Class C β-lactamases	[72,77]
bla-TEM	Class A β-lactamases	[77]
blaACT-MIR	Class C β-lactamases	[77]
blaCMY-2	Class C β-lactamases	[77]
blaCTX-M, blaCTX-M-1, blaCTX-M-9, CTX-M-32	Class A β-lactamases	[77]
blaDHA	β-lactamases	[77]
blaFOX	β-lactamases	[77]
blaGES	β-lactamases	[77]
blaOXA, blaOXA-48, blaOXA-58, intl1	Class D β-lactamases	[44,77]
blaSHV	Class A β-lactamases	[77]
blaVIM-1	Imipenem, Class B (metallo-)β-lactamases	[72,77]
ermB	Erythromycin, Macrolides	[72]
ermF	Clindamycin, Streptogramins	[77]
mcr-1	Colistin	[44]
nptII (aph(3')-IIa), nptIII (aph(3')-IIIa)	Aminoglycoside	[71]
qacEΔ1	Quaternary ammonium compounds, resistance to antiseptics	[53,77]
qnrA	Fluoroquinolones	[64]
sul1, sul2, sul3	Sulfonamides	[64,66]
tet(A), tet(B), tet(L), tet(M), tet(O), tet(S), tet(W), tet(X)	Tetracyclines	[64,77]
vanA	Vancomycin, Glycopeptides	[72,77]

2. Included literature

2.1. Results of the searches and study selection

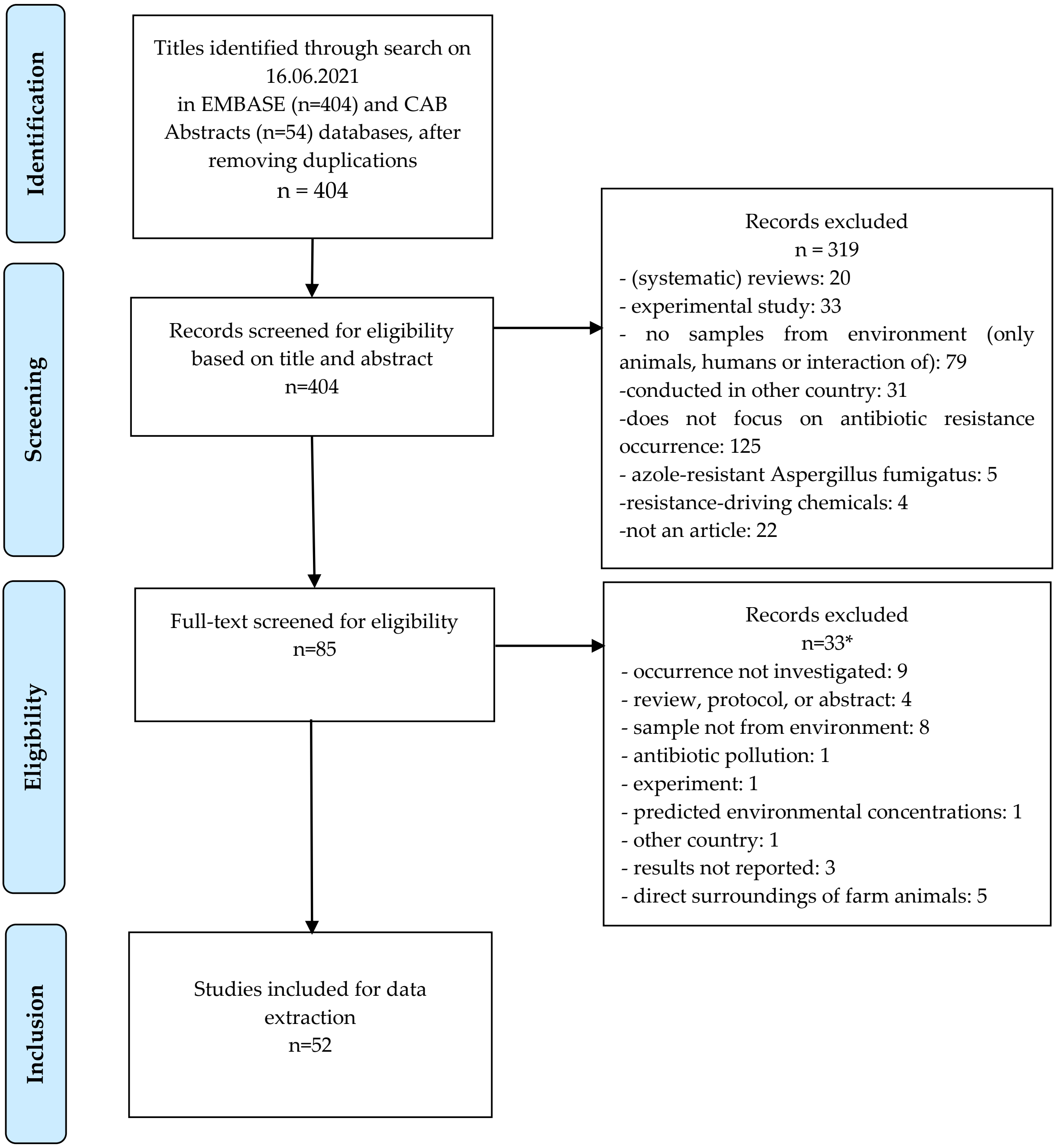

Figure S1. Results of the searches and study selection represented following the Prisma chart

*33 records which were excluded after the full-text screening based on the selection criteria are reported in section 2.5 of this supplement

2.2. *Overview of the included studies*

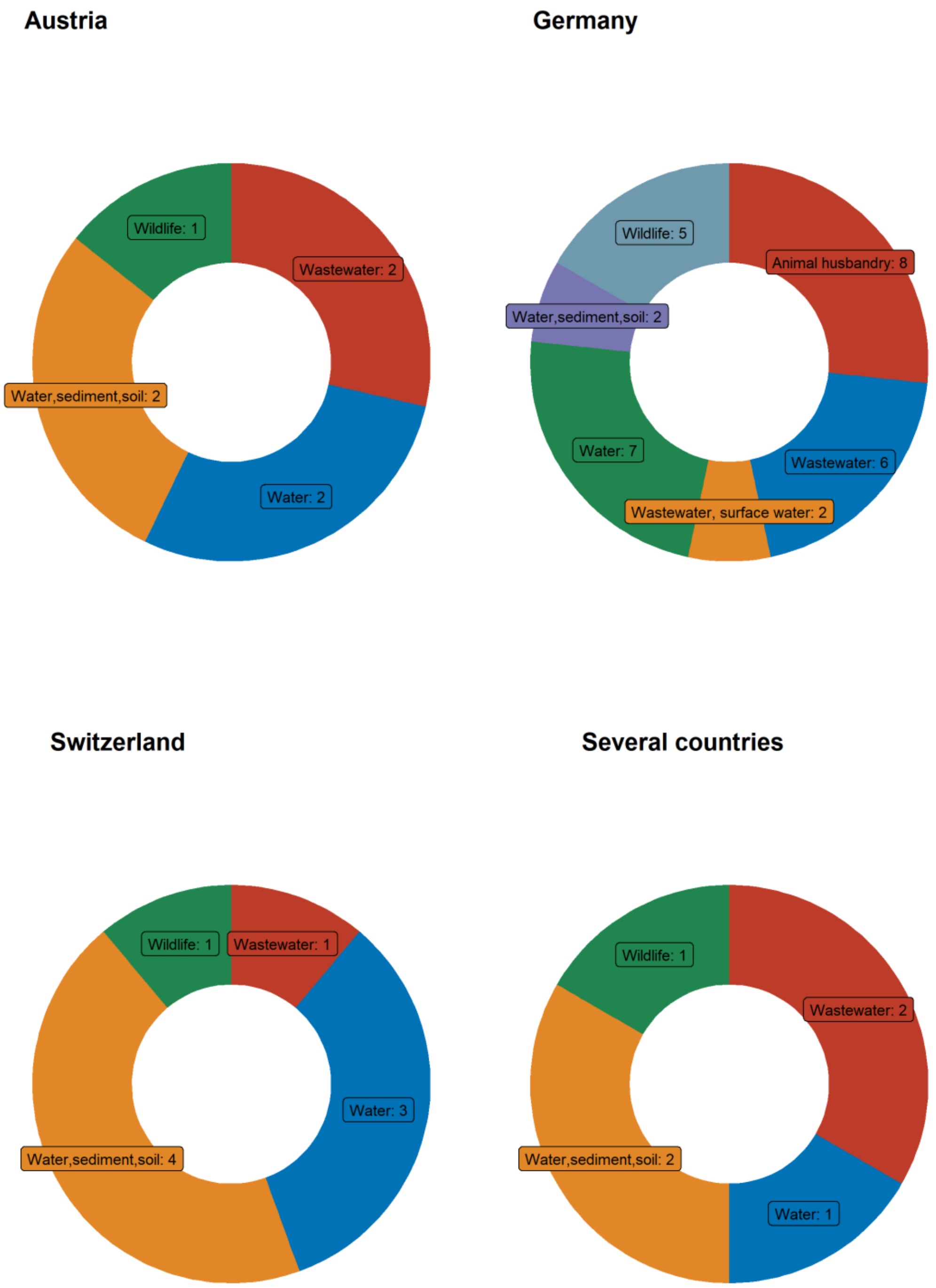

Figure S2. Number of studies by country and environment

2.3. *Overview of the sampling strategies*

In Germany, several environmental reservoirs were examined, including water, wastewater, wildlife, and animal husbandry. Two studies looked at different sources such as food chains and several environmental sources [72,73]. Out of 13 studies conducted between 2015 and 2020, seven examined water and wastewater, three studies analyzed wildlife, including wild boars, roe deer, wild ducks and geese, flies, and one each water and sediment, wastewater from a farm, food chain and environmental sources. Nine studies conducted sampling campaigns between 2010 and 2015 investigating water, animal husbandry, and wildlife. Out of five studies conducted before 2010, three investigated manure, one looked at wild rodents, and one at surface water. Additionally, three studies also investigated water sources but did not report the sampling period.

In Switzerland, mainly water and sediment were examined. Among the studies, which conducted sampling in 2015-2020, two studies examined water, one study analyzed sediment, and one study looked at wildlife, particularly wild birds. Among the earlier conducted studies (n = 3), water and wastewater samples were analyzed. Two studies that investigated water and sediment did not report the sampling period.

In Austria, also mostly water was examined. Four studies reported analyses of water and wastewater. One study examined water and soil, one investigated soil only, and one studied two populations of rooks. All studies reported the sampling period. Three conducted sampling between 2010 and 2015, two in 2015-2020 and two before 2010.

2.3.1. Antibiotic resistance in water sources.

In Germany and Austria, the waters of rivers Rhine [29,31], Danube [25,31], Glan [25], Inn [25], Traun [25], Swist [27], Lahn [24], Weser [28], and Mur [34] were sampled for resistance-related analyses. Among these seven studies, only two studies conducted a relatively broad investigation of antibiotic resistance in surface water. Stoll et al. [31] studied the presence of antibiotic resistance genes against eight antibiotic classes in rivers Rhine and Danube. In addition, Stange et al. [29] analyzed the waters of river Rhine to detect the presence of antibiotic resistance genes against ten antibiotic compounds, coliform and *E. coli* bacteria which indicated fecal pollution in the aquatic environments. The other five studies conducted more targeted analyses. Schreiber et al. [27] explored resistance of environmental Rhodospirillaceae to antibiotics used in human medicine in the waters of river Swist in Germany. Lepuschitz et al. [25] aimed to identify methicillin-resistant Staphylococcus aureus (MRSA) in the waters of rivers Danube, Glan, Inn and Traun in Austria. The presence of blaCTX-M as a potential indicator of resistance spread in the waters of river Lahn in Germany was investigated by Herrig et al. [24]. Zarfel et al. [34] screened the waters of river Mur in Austria for the presence of extended-spectrum beta-lactamases and carbapenemases harboring Enterobacteriaceae. Another study [28] looked at microplastics as a potential mode of spread of multidrug-resistant (MDR) E. coli in the water of river Weser in Germany.

The bathing waters were analyzed in a study conducted in North Rhine-Westphalia [23] in Germany. Döhla et al. [23] conducted a broader analysis of antibiotic resistance, aiming to identify the presence of ARBs and ARGs in bathing waters.

Three studies in Switzerland investigated waters of lakes and inland streams [22,26,33] with a focus on the presence of carbapenemase-producing Enterobacteriaceae [22], antibiotic resistance patterns of E.coli [33], and qnr genes among Aeromonas spp. strains [26]. Spring and drinking waters were analyzed in two studies in Germany [30,32], which investigated the presence of clinically relevant ARBs and ARGs.

2.3.2. Antibiotic resistance in wastewater

Agrawal et al. [35] compared the occurrence and fate of ARGs between a pond system in Namibia and an advanced activated sludge system in Germany. In another German study, Alexander et al. (2020) [36] investigated daily discharges of different ARGs and bacteria in effluents released from several WWTP clusters, including communal, food production, slaughter house wastewater, and hospital discharge. Alygizakis et al. [37] overviewed effluent wastewater discharged into the Danube River and its tributaries and conducted the sampling in multiple sites in nine different countries, including Austria

and Germany. Bäumlisberger et al. [38] analyzed the presence of antibiotic resistance genes in wastewater upstream and downstream from a non-hospital medical care facility, i.e. a nursing home located in southwest Germany. Klees et al. [73] explored the spread of multidrug-resistant bacteria at different stages of food production and in the environment and quantified the prevalence of various resistance profiles of the included pathogens across different sources, including sludge and sewage. Alexander et al. (2015) [72] investigated the dissemination of ARGs and opportunistic bacteria in different water environments, including hospital wastewater and municipal WWTP.

Biggel et al. [39] focused on resistance against fosfomycin (critically important phosphonic acid derivative). They examined wastewater in Switzerland to study the complete sequences of fosA-carrying plasmids isolated from E. coli and Klebsiella spp. coming from food and environmental sources. In Austrian studies, Galler et al. [40] screened for multidrug-resistant pathogens in activated sludge, and Reinthaler et al. [43] evaluated resistance patterns of E. coli isolated from samples of sewage, sludge and receiving waters from three sewage treatment plants. In other four German studies, wastewater samples were examined for the presence of various resistance patterns. Hembach et al. [41] screened wastewater from seven WWTPs different in their catchment area and population for the presence of resistance gene mcr-1, which mediated resistance against colistin (critically important of highest priority polymyxin). Schages et al. [44] also looked at mrc and beta-lactamase genes in wastewater. They studied the seasonal variation of the gene concentrations in sewage sludge and effluent water of a secondary WWTP. Müller et al. [42] investigated the presence and the spread of multidrug-resistant and extensively drug-resistant (defined as resistant to at least one antibiotic in all but two or less antibiotic classes) carbapenemase-producing Gram-negative bacteria originated from hospitals to wastewater. The researchers analyzed wastewater discharged from hospitals, inflow to a receiving municipal WWTP and WWTP effluent, receiving river water upstream and downstream from WWTP. Therefore, they explored possible dissemination of the bacteria from hospital buildings to surface waters in the area. Schmiege et al. [45] also explored the presence of multidrug-resistant ESBL-producing E. coli in community wastewater which stemmed from socio-spatially different districts.

2.3.3. Antibiotic resistance in the environment of animal husbandry

Wolters et al. [53] evaluated the connection between antibiotic use and antibiotic residues to the presence of resistance genes and mobile genetic elements in organic fertilizers obtained from pig manures. Savin et al. [50] investigated the occurrence and characteristics of antimicrobial-resistant bacteria in the different types of wastewater from poultry husbandry and processing.

Binh et al. [46] analyzed samples of piggery manure originating from 15 different pig farms (nationwide) and explored the presence and types of plasmids that can function as shuttles of antibiotic resistance genes.

Two studies [47,48] used bacteria obtained from the samples previously collected within a national ESBL-surveillance project RESET (www.reset-verbund.de), which examined pig farms in Germany. The samples included environmental swabs, manure, flies, mice feces, and dogs feces. Fischer et al. [47] retrospectively looked at the spread of blaVIM-1 positive isolates of E. coli and Salmonella Infantis in carbapenemase-positive pig farms. Guenther et al. [48], in turn, studied the distribution of mcr-1-harboring E. coli in the environment of pig farms.

Two studies were conducted in the Bavaria region. Hölzel et al. [49] investigated the presence and antibiotic resistance of Salmonella spp. in liquid manure in pig farms. Schwaiger et al. [52] also analyzed liquid manure samples from pig farms to describe how contamination of the environment with antibiotics influences the occurrence of phenotypic and genetic resistance.

Schoenfelder et al. [51] conducted a study in North Rhine-Westphalia and Lower Saxony regions using dust and manure samples. The authors screened for coagulase-negative staphylococci, methicillin- susceptible Staphylococcus aureus (MSSA) and MRSA to assess their potential for harboring antibiotic resistance genes.

2.3.4. Antibiotic resistance in wildlife

Within a monitoring program, Plaza-Rodríguez et al. [57] investigated the presence and resistance of five bacterial species in wild boars, roe deer, wild ducks and geese in Germany on the nationwide scale. The targeted bacterial species included *Salmonella* spp. (in wild boars and wild ducks and geese),

Campylobacter spp. (in roe deer and wild ducks and geese), Shiga toxin-producing *E. coli*, commensal *E. coli* and ESBL- or ampicillinase class C beta-lactamase-producing *E. coli* in all animals, and MRSA in wild boars. The study war incorporated within an existing program. The geographical location of origin of the animals was not reported. The types of samples varied and included fecal samples, nasal swabs, and cadaver samples. The resistance of the isolates was tested against 14 substance classes: gentamicin, chloramphenicol, cefotaxime, ceftazidime, nalidixic acid, ciprofloxacin, ampicillin, colistin, sulfamethoxazole, trimethoprim, tetracycline, azithromycin, meropenem, tigecycline.

Other studies considered one animal species only and provided more restricted analyses. One study in each country, Austria, Germany and Switzerland, investigated wild birds. In Austria, Loncaric et al. [56] examined migratory and resident populations of rooks (*Corvus frugilegus*) as a potential indicator of the spread of antibiotics and resistance in their habitats. The authors screened for the occurrence of beta-lactamase-producing *Enterobacteriaceae*, MRSA and cefotaxime-resistance in the fecal samples. In Switzerland, Zurfluh et al. [61] analyzed fecal samples of wild birds admitted to a care center for the presence of antibiotic-resistant *E. coli* with resistance to third-generation cephalosporins, carbapenems, aminoglycosides, and colistin. In Germany, Schaufler et al. [59] looked at the presence of clinically relevant ESBL-producing *E. coli* in wild birds in Berlin. The authors investigated the cloacal swabs of rescued birds of around 40 different species.

Other three studies conducted in Germany examined one each wild boars, wild rodents and flies. Reinhardt et al. [58] investigated the tonsils of hunted wild boars for the occurrence and resistance of *Yersinia pseudotuberculosis*. Eight wild rodent species and one shrew species in rural areas were examined for the presence of antibiotic-resistant *E. coli* in the study by Guenther et al [54]. The isolates were tested for resistance against beta-lactams, tetracyclines, aminoglycosides and sulfonamides. Wetzker et al. [60] also examined the presence of ESBL-producing *E. coli* but used flies collected in an urban center as an indicator.

Loncaric et al. [55] examined the presence of beta-lactamase-producing and fluoroquinolone-resistant *Enterobacteriaceae* in fecal samples of the European mouflons (*Ovis orientalis musimon*) in Austria and Germany.

2.3.5. Antibiotic resistance in sediment and soil

Four Swiss studies examined sediment samples in a more general explorative approach [63,64,66,68]. Czekalski et al. (2012) [63] investigated samples from different sources obtained in Vidy Bay, Lake Geneva, including sediment close to a WWTP and drinking water pump, wastewater effluents and freshwater. The authors conducted this study to assess whether WWTP can provide an environment for selecting multidrug-resistant bacteria and horizontal transfer of resistance. In a later study, Czekalski et al. (2014) [64] analyzed samples of sediment cores at 22 sites of Lake Geneva to investigate the spatial distribution and abundance of different ARGs, such as *sul*1, *sul*2, *tet*(B), *tet*(M), *tet*(W) and *qnrA*. Thevenon et al. [68] looked at Vidy Bay and the Creux de Genthod region and examined sediment samples originating from a site close to WWTP outlet pipes and coast. The authors aimed to quantify the presence and distribution of multidrug-resistant bacteria, fecal indicator bacteria, and ARGs. Laffite et al. [66] looked at the sediment of another freshwater lake in Switzerland, Lake Brêt, and evaluated the concentration of beta-lactam and sulfonamide resistance genes: *bla*$_{TEM}$, *bla*$_{SHV}$, *bla*$_{CTX-M}$, *bla*$_{NDM}$, *sul*1, and *sul*2. In Germany, Reichert et al. [67] evaluated different strategies to monitor the presence of antibiotic-resistant bacteria in rivers. For the study, the authors took different samples, including surface water, sediment, and biofilm, and analyzed them for 13 ARGs and gene markers of facultative pathogenic bacteria. The other two studies had a more focused approach. Devarajan et al. [65] examined the spread of antibiotic-resistant *Pseudomonas spp*. in sediments of aquatic environments in Switzerland, Democratic Republic of the Congo and India. In a German study, Bier et al. [62] examined *Vibrio vulnificus* and *Vibrio cholerae non-O1/non-O139* isolates obtained from coastal water, sediment, bivalve molluscs of the Baltic Sea and North Seas for antimicrobial resistance patterns.

Three studies that investigated soil conducted comparatively specific analyses. Malik et al. [70] in the samples of soil obtained from Berlin Tiergarten, an urban park, and an abandoned sewage field in Berlin and India and quantified the presence of plasmids of incompatibility groups: IncP, IncN, IncW, and IncQ. The other two studies were conducted in Austria. Woegerbauer et al. [71] sampled the soil from 50 maize fields and 50 potato fields and analyzed the presence of *aph*(30)-IIa/nptII and *aph*(30)-

IIIa/nptIII ARGs, which are associated with plant biotechnology and resistance to aminoglycosides. Linke et al. [69] investigated the occurrence of *Listeria monocytogenes* in the samples of water and soils with different compositions.

2.4. Results tables

Table S2. Overview of the included studies (n=52).

First author	Year	Country	Locality	Research focus	Environmental source	Sampling period
Agrawal et al.[35]	2020	Germany (and Namibia)	not reported	Occurrence and fate of ARGs	WWTP (activated sludge system)	not reported
Alexander et al.[36]	2020	Germany	not reported	Daily discharges of ARGs and FPB in effluents	Conventionally treated WWTP effluents	Feb - Nov 2018
Alexander et al. [72]	2015	Germany	not reported	Dissemination of ARGs and opportunistic bacteria in different anthropogenically influenced aquatic habitats	Hospital wastewater, municipal WWTP (influent, effluent), river surface water, rain overflow basins, groundwater	2 years, not reported
Alygizakis et al. [37]	2019	9 countries including Germany and Austria	The Danube River Basin. Germany: Augsburg. Austria: Amstetten	Overview of effluent wastewater released into the Danube River	WWTP on the Danube River Basin, mainly of industrial wastewater	Aug, Sep 2017
Bäumlisberger et al. [38]	2015	Germany	South-west Germany	Taxonomic composition and content of resistance determinants in wastewater	Non-hospital medical care facility wastewater	Nov 2012 - Mar 2013
Bier et al. [62]	2015	Germany	Baltic Sea and North Sea	Resistance of *Vibrio vulnificus* and *Vibrio cholerae* non-O1/non-O139	Coastal water, sediment, bivalve molluscs	2004 - 2012; 2009 - 2014
Biggel et al. [39]	2021	Switzerland	multiple	Complete sequences of 13 fosA1 plasmids, fosfomycin resistance	A hospital-associated WWTP; rural and urban surface water	2012 - 2019
Binh et al. [46]	2008	Germany	Nationwide	Occurrence and types of plasmids functioning as shuttles of ARGs	Piggery manure; manure storage tanks of 15 farms	May - Jun 2006
Bleichenbacher et al. [22]	2020	Switzerland	not reported	Occurrence in river water	Rivers, inland canals, and streams	May - Aug 2019
Czekalski et al. [64]	2014	Switzerland	Vidy Bay, Lake Geneva	Spatial distribution of different ARGs	Freshwater lake sediments	Aug 2011

Czekalski et al. [63]	2012	Switzerland	Lake Geneva Vidy Bay, Lausanne	WWTP as a barrier for MRB in the wastewater stream or as an environment for selection of MRB and horizontal transfer of resistance factors. Role of the lake as a potential reservoir of ARB and ARG	Hospital and municipal raw sewage, treated effluent from Lausanne's WWTP, lake water, sediment samples close to WWTP and close to a drinking water pump	not reported
Devarajan et al. [65]	2017	Switzerland (Democratic Republic of the Congo, India)	Vidy Bay, Lake Geneva	Antibiotic resistant *Pseudomonas spp*. in the aquatic environment in different climate conditions: tropical and temperate	Sediments	May 2012
Döhla et al. [23]	2020	Germany	North Rhine-Westphalia	Presence of resistance in bathing water, development of a tool to support prevention measures	Bathing waters	May - Sep 2018
Fischer et al. [47]	2017	Germany	not reported	Distribution of *VIM-1*-positive isolates in any of the carbapenemase-positive swine farms	Environmental swabs, manure, flies, mice faeces, farmers dogs' faeces	2011-2012
Galler et al. [40]	2018	Austria	Area of Graz, Styria	Multi-resistant pathogens in WWTP	Activated sludge	Sep 2011 - Feb 2012
Guenther et al. [54]	2010	Germany	Bavaria, Mecklenburg-Pomerania, Saxony-Anhalt, Saxony and Brandenburg	Antimicrobial resistance status of *E. coli* in wild small mammals in rural areas (a pilot study)	Wild rodents in rural areas	2007 - 2008
Guenther et al. [48]	2017	Germany	not reported	Environment surrounding the farms as source for mcr-1-positive strains	Boot swabs, flies, dog faeces and manure	2011 - 2012
Hembach et al. [41]	2017	Germany	not reported	Presence of the colistin resistance gene mcr-1	WWTP	not reported
Herrig et al. [24]	2020	Germany	Lower Lahn River	ARG dynamics in effluent receiving surface waters and the contribution of faecal pollution sources	Surface water	Oct 2011 - Dec 2012

Hölzel et al. [49]	2008	Germany	Bavaria	Presence of *Salmonella spp*. in liquid manure	Manure samples	Autumn 2002 - Spring 2003
Klees et al. [73]	2020	Germany	East Westphalia-Lippe	Presence of MRB within the meat production chain	4 stages of meat production: slaughterhouses, meat-processing plants, fresh food products and the urban environment	2018 - 2019
Laffite et al. [66]	2020	Switzerlan d	Lake Brêt	Impact of the catchment agricultural practices on the accumulation of ARGs in surface sediment	Sediments from Lake Brêt used as drinking-water reservoir	Sep 2017
Lepusch itz et al. [25]	2018	Austria	four rivers: Danube, Glan, Inn, Traun	Presence of MRSA USA300 strain in river water, phylogenetic relatedness to clinical CA-MRSA USA300 isolates	Surface water, bathing water	2016
Linke et al. [69]	2014	Austria	Rax, Regelsbrunn	Occurrence of *L. monocytogenes* in soil from areas with different soil compositions	Soil and water	2007 - 2009
Loncaric et al. [55]	2016	Austria, Germany	Öttingen, Kösingen (Germany); Vienna, Miesenbach, Rohr-Gebirge, Orth an der Donau, Schützen, Klein-Rust, Eggenburg, Zell-See (Austria)	Presence of beta-lactamase producing or fluoroquinolone-resistant *Enterobacteriaceae* in European mouflons	Mouflons (*Ovis orientalis musimon*)	Mar 2012 - Jan 2016
Loncaric et al. [56]	2013	Austria	Lobau and Wulkaprodersdorf	Rooks (*Corvus frugilegus*) as indicators for the potential circulation of antibiotics in the native habitat	Rooks (*Corvus frugilegus*) 2 populations: migratory and a resident population.	Mar 2013
Malik et al. [70]	2008	Germany, India	The urban park Berlin Tiergarten and the abandoned sewage field Berlin-Buch	Detection of plasmid specific sequences from prevalent plasmid Inc groups and on the detection of the antibiotic resistance gene pool in wastewater irrigated soils	Soil under anthropogenic influences and soil which used to be under wastewater application	not reported
Müller et al. [42]	2018	Germany	North-Rhine Westphalia, small river in the western part of Germany	Spread of MDR and XDR Gram-negative bacteria from hospitals to environment	Clinical/urban wastewater, surface river water	Sep 2016 -Jul 2017

Picão et al. [26]	2008	Switzerland	The southern part	Spread of *qnr* genes among *Aeromonas spp*. In rivers and lakes in the Swiss Alps	Lake water	2002 - 2005
Plaza-Rodríguez et al. [57]	2020	Germany	Nationwide	Antibiotic resistance patterns in wild animals	Wild boars (*Sus scrofa*), roe deer (*Capreolus capreolus*), wild ducks (family *Anatidae*, subfamily *Anatinae*) and geese (family *Anatidae*, subfamily *Anserinae*)	2016 (wild boar), 2017 (roe deer), 2019 (wild ducks and geese)
Reichert et al. [67]	2021	Germany	The Kraichbach River, Baden-Württemberg	Different monitoring strategies for antibiotic-resistant bacteria in rivers	WWTP-impacted river in surface water, sediment, and biofilm. The river is the effluent receptor of 6 WWTPs	Feb - Jun 2019
Reinhardt et al. [58]	2018	Germany	Brandenburg, Mecklenburg-Western Pomerania	Prevalence of *Yersinia pseudotuberculosis* in wild boars	Wild boars	Feb 2015 - Dec 2016
Reinthaler et al. [43]	2003	Austria	Southern Austria	Resistance patterns of *E. coli* in WWTPs	Sewage, sludge and receiving waters from 3 different sewage treatment plants	Apr-Sep 2000
Savin et al. [50]	2020	Germany	not reported	Occurrence and characteristics of ARB in the wastewater of poultry slaughterhouses	Process water from delivery and unclean areas, wastewater from the in-house WWTP	Dec 2016 - Sep 2018
Schages et al. [44]	2020	Germany	District of Kleve	Impact of temperature on the variation of beta-lactamase and *mcr* genes in a WWTP	Wastewater, sewage sludge and effluent water; secondary WWTP	May 2018 - Apr 2019
Schaufler et al. [59]	2016	Germany	Berlin	Occurrence of clonal or closely related antimicrobial-resistant clinically relevant *E. coli* in wild birds	Rescued wild birds	2011 - 2014
Schmiege et al. [45]	2021	Germany	The Ruhr Metropolis	Presence of MRGN and ESBL-producing *E. coli* in community wastewater from socio-spatially different districts	Community wastewater, in-patient health care facility, WWTP	Apr 2019-Mar 2020
Schoenfelder et al. [51]	2017	Germany	North Rhine-Westphalia, Lower Saxony	Potential of coagulasenegative staphylococci (CoNS) and *S. aureus* as putative reservoirs for ARGs	Dust and manure samples	Feb 2013 - Sep 2013

Schreiber et al. [27]	2013	Germany	River Swist	Resistance of environmental *Rhodospirillaceae* to antibiotics used in human medicine	Wastewater samples; water samples from upstream and downstream from STP sites, water samples from tributary	not reported
Schwaiger et al. [52]	2009	Germany	Bavaria	Impact of antibiotic contaminated environment on microorganisms on both a phenotypic and a genetic level	Liquid manure samples from pig farms	Autumn 2002 - Spring 2003
Song et al. [28]	2020	Germany	River Weser	Colonization of synthetic and natural particles by ESBL-producing *E. coli*	Surface water	Jul - Sep 2018
Stange et al. [29]	2016	Germany	River Rhine	Occurrence of ARGs, integrons and virulence genes in coliform and *E. coli* bacteria in river water	Surface water	Jun 2006 - Jun 2007
Stange et al. [30]	2020	Germany	South-western Germany, Gallusquelle	Microbiological contamination of the spring water	Ground water, spring water	Mar 2012 - Jun 2013
Stoll et al. [31]	2012	Germany (and Australia)	Rivers Rhine and Danube	Occurrence of ARGs in river waters	Surface water	not reported
Thevenon et al. [68]	2012	Switzerland	Lake Geneva: The Bay of Vidy and the Creux de Genthod region	Presence and distribution of faecal indicator bacteria and ARGs	Sediment	not reported
Voigt et al. [32]	2020	Germany	Drinking water reservoir	Occurrence in surface water	Drinking water	Aug 2018 - Jun 2019
Wetzker et al. [60]	2019	Germany	Berlin, urban centre	Data on the current prevalence of ESBL-producing *E. coli* in flies	Flies	July 2016
Wicki et al. [33]	2011	Switzerland	North-western, Lützel stream	Faecal contamination of spring water, localizing possible input sites for contaminants	Spring water, surface water	Mar - Sep 2009
Woegerbauer et al. [71]	2015	Austria	Maize and potato growing regions	Presence of *aph(30)-IIa/nptII* and *aph(30)-IIIa/nptIII* genes	Agricultural soils	Aug - Sep 2011
Wolters et al. [53]	2016	Germany	Lower Saxony	Link between antibiotic usage and antibiotic residues to ARGs and mobile genetic elements in organic fertilizers	Pig manures, 8 pig fattening and 6 pig breeding farms. Digestates from 8 biogas plants	Autumn 2012
Zarfel et al. [34]	2017	Austria	The River Mur (Graz)	Presence and diversity (on the genetic and phenotypic levels)	Surface water	Oct 2015 - Jan 2016

Zurfluh et al. [61]	2019	Switzerlan d	Sempach	*Enterobacteriaceae* with transmissible resistance to third-generation cephalosporins, carbapenems, aminoglycosides, and colistin in wild birds	Wild birds admitted to the care centre, the rehabilitation centre	May - Oct 2018

Table S3. Presence of antibiotic resistance or antibiotic resistance genes in water bodies as reported in the included studies (n = 13)

Source	Samples	Target	Reported ARB, ARGs presence	Refs
Locality: not reported. Source: Rivers, inland canals, and streams	Period: May-Aug 2019. Sampling: 500 mL surface samples. Number of samples: 164. Number of isolates: not reported	carbapenemase producing *Enterobacteriaceae* (CPE)	CPE included 12 *E. coli*, one *Citrobacter freundii*, one *Enterobacter kobei*, one *Klebsiella aerogenes*, one *Klebsiella variicola*, one *Raoultella ornithinolytica* strain. 17 (10%) of 164 are CPE. 15 (88%) of the strains were MDR. 14 (82%) and 10 (59%) of the strains resistant to the 3rd and 4th generation cephalosporins cefotaxime and cefepime, respectively	Bleichenbacher (2020). Switzerland [22]
Locality: North Rhine-Westphalia, 16 sampling sites of bathing waters in the region. Source: Bathing waters	Period: May-Sep 2018. Sampling: 750 ml of water Number of samples: 64. Number of isolates: not reported	*E. coli*, *Pseudomonas aeruginosa*	3 (4.68%) samples contained *E. coli* and *Pseudomonas aeruginosa*. *E. coli* - multidrug resistant. *Pseudomonas aeruginosa* - colistin-resistant	Döhla (2020). Germany [23]
Locality: lower Lahn River, two sites with different degrees of wastewater effluent impact. Source: Surface water	Period: Oct 2011-Dec 2012. Sampling: Grab samples, 100 mL to 250 mL. Number of samples: 97. Number of isolates: not reported	*E. coli*, blaCTX-M ARG	blaCTX-M genes encoding resistance to beta-lactam antibiotics: cephalosporins, carbapenems and penicillins in *Enterobacteriaceae*. blaCTX-M ARG were detected in all water samples	Herrig (2020). Germany [24]
Locality: four rivers Danube, Glan, Inn, Traun. Source: Surface water, bathing water	Period: 2016. Sampling: 100 ml, 20 to 30 cm below the river surface and 50 to 100 cm apart from the river bank. Number of samples: 12. Number of isolates: not reported	MRSA USA300	1 sample was identified as MRSA USA300 strain. Resistant to beta-lactams (benzylpenicillin, amoxicillin-clavulanic acid, cefoxitin), fluoroquinolones (ciprofloxacin, moxifloxacin) and erythromycin	Lepuschitz (2018). Austria [25]
Locality: The southern part, lakes. Source: Water	Period: 2002 - 2005. Sampling: Water samples. Number of samples: not reported. Number of isolates: not reported	qnrS	1 isolate identified qnrS gene in *Aeromonas allosaccharophila*. Gene resistant to quinolones	Picão (2008). Switzerland [26]
Locality: Catchment of the river Swist; five sewage treatment plants located in the catchment area; sites with different levels of	Period: not reported. Sampling: 10 cm depth every 2 weeks over the period of a hydrological year. Number of samples: not reported. 614 isolates	*Rhodospirillaceae*	MDR occurred more often than resistance to a singled antibiotic. Highest MDR level was detected in the tributaries. 46% of MDR to 4 antibiotics in *Rhodopseudomonas palustris* isolates. Resistance to ofloxacin (22%), ceftazidime (22%) and piperacillin	Schreiber (2013). Germany [27]

human impact on the environment. Source: wastewater samples; water samples from upstream and downstream from WWTP sites, water samples from tributary			(28%), cephazoline (36%), oxacillin (41%). Resistance differed between the sampling sites	
Locality: four sites along the River Weser: Bremen, Brake, Bremerhaven, Helgoland. Source: Surface water	Period: Jul-Sep 2018. Sampling: Surface water was collected with a bucket. Number of samples: 9. Number of isolates: not reported	*E. coli*	64% resistant to fluoroquinolones, sulfamethoxazole/trimethoprim, aminoglycosides additionally to broad spectrum penicillins and cephalosporins. 31% MDR *E. coli* was isolated from the Helgoland site (highest MDR prevalence). 57% of MDR isolates were resistant to fluoroquinolone; 12% of MDR resistant to aminoglycosides and 19% to sulfamethoxazole/trimethoprim. 36 (36.3%) isolates - ESBL only; 32(32.3%) -resistant to fluoroquinolone only; 6 (6.1%) - resistant to aminoglycosides only	Song (2020). Germany [28]
Locality: River Rhine. Source: Surface water	Period: Jun 2006-Jun 2007. Sampling: Grab samples. Number of samples: 17. 100 isolates	Coliform bacteria, *E. coli*	Isolates of coliform bacteria: overall resistance (resistance to at least one antibiotic) was 54%. Of the resistant isolates, 41.3% was *E. coli*, 33.3% was *Klebsiella*, 16.7% could not be allocated to specific genera. Amoxicillin (48%), tetracycline (9%), sulfamethoxazole/trimethoprim (11%)	Stange (2016). Germany [29]
Locality: South-western Germany, Gallusquelle. Source: Ground water, spring	Period: March 2012 - June 2013. Sampling: Grab samples, 1.5-liter, 30 cm below the water surface. Number of samples: 76. Number of isolates: not reported	*E. coli* and *enterococci*	Of the samples: 39.5% sul2 genes, sul1 - 36.8%, dfrA1 - 30.3%, dfrA12 - 11.8%, tet (C) - 40.8%, tet(B) - 25.0%, tet(A) - 6.6%, tet(K) 5.3%; 42.1% were positive for ermB, aadA - 17.1%. 48.7% of sample have tetracyclines resistance genes (efflux genes), sulfonamide resistance genes were frequently detected; trimethoprim resistance genes in 41.8% of the samples; macrolide resistance genes in 42.1% of the samples; aminoglycoside resistance gene in 17.1% samples	Stange (2020). Germany [30]
Locality: Rivers Rhine and Danube. Source: Surface water	Period: not reported. Sampling: Duplicate grab samples, 500 mL each. Number of samples: 43.	Coliform bacteria, *E. coli*	blaSHV 16%, ampC 19%, blaPSE-1 gene was not detected, tet(A) 7%, tet(B) 16%, tet (C) 22%, sul1 98%, sul2 77%, dfrA1 43%, dfrA13 gene was not detected, aac(3)-Iia 5%, cat1 5%, cat2 9%, floR 3%, ermB 68%,	Stoll (2012). Germany (and Australia),

	Number of isolates: not reported		ermA gene was not detected, vanB 2.3%. Of the samples, 100% sulfonamide resistance genes; 45% tetracycline resistance genes; 55% trimethoprim resistant; 16% beta-lactam resistance, 16% chloramphenicol resistance; 67% macrolide resistance genes; aminoglycoside and glycopeptide resistance genes in <10%	only for Germany reported here [31]
Locality: Drinking water reservoir, 7 sites; is subject to the discharge of treated WW from two municipal STPs which do not treat WW from sources like hospitals or slaughterhouses. Source: Drinking water	Period: August 2018 - June 2019. Sampling: Grab samples; 250 mL. Number of samples: 48. 113 different isolates	Clinically relevant ARB: *P. aeruginosa*, *E. coli*, A. calcoaceticus-baumannii (ACB) complex, *E. faecium* and other *Enterobacteriales* (including *Klebsiella* spp. *Citrobacter* spp. and *Enterobacter* spp.), MRSA	Acinetobacter calcoaceticus-baumannii complex 18.3%; 1 isolate MDR ACB complex; *E. coli*: 11 of 30 bacterial isolates were MDR; *Enterobacteriales* (predominately Klebsiella spp. and Enterobacter spp.) 12 of 24 isolates were MDR; *P. aeruginosa* no MDR; *E. faecium*: 10 out of 11 were MDR. ACB complex MDR resistance towards piperacillin/tazobactam (P/T), cefotaxime (CEFO), ceftazidime (CEFTA) and trimethoprim-sulfamethoxazole (T/S); *E. coli* resistant to CEFO, CEFTA, ciprofloxaci, and T/S; All *Klebsiella* isolates were resistant for CEFO and CIP. *Enterobacter* spp. resistance pattern varied widely including the substances P/T, CEFO, CEFTA, CIP, CHL, T/S and fosfomycin. Prevalence not reported. MRSA was not detected	Voigt (2020). Germany [32]
Locality: North-western, Lützel stream, downstream from WWTP. Source: Spring water, surface water	Period: March-Sep 2009. Sampling: 1,000, 100, and 10 ml for spring water and 100, 10, and 1 ml for surface water; 15 to 20 cm depth. Number of samples: Spring water: 22; Surface water:16. Number of isolates: not reported	*E. coli*	Multidrug resistant *E. coli* were detected in all surface water samples and in 14 (63.6%) spring water samples.	Wicki (2011). Switzerland [33]
Locality: The River Mur (Graz); one sampling site; upstream of the urban WWTP. Source: Surface water	Period: Oct 2015-Jan 2016. Sampling: Iterative sampling; 500 ml; 30 cm below the river surface, 50 cm apart from the river bank. Number of samples: 4 samples. 109 isolates	ESBL- and carbapenemases harboring *Enterobacteriaceae*	Enterobacteriaceae: *E. coli* and *Klebsiella oxytoca*; 71 isolates (65.1%) were identified as ESBL-positive; 2 isolates (1.8%) were tested positive for carbapenemases. *K. oxytoca* revealed resistance to piperacillin, amoxicillin/clavulanic acid, cephalexin, cefuroxime, ceftazidime and nalidixic acid.	Zarfel (2017). Austria [34]

			70 E. coli ESBL isolates were resistant to ampicillin, piperacillin and cefuroxime. 50% *E. coli* ESBL were non-beta-lactame antibiotic and trimethoprim co-resistant; nalidixic acid 35.71%, tetracycline 44.29%, trimethoprim/sulfamethoxazole 44.29%; 10.00% resistance to fluoroquinolones.	

Table S4. Presence of antibiotic resistance or antibiotic resistance genes in wastewater as reported in the included studies (n = 13)

Source	Samples	Target	Reported ARB, ARGs presence	Refs
Locality: not reported. Source: WWTP (activated sludge system (AS))	Period: not reported. Sampling: Samples from the influent and effluent; 3 grab samples each; 5L. Number of samples: 6. Number of isolates: not reported	ARGs: Targeted metagenomic analysis	Pathogens (ARB not reported): Proteobacteria in the AS influent (40%) and effluent (26%). Firmicutes (30%) > Bacteroidetes (12%)> Actinobacteria (8%) in the AS influent; and Actinobacteria (8%)> Patescibacteria (23%)> Bacteriodetes (9%)> Firmicutes (5%) in the AS effluent.<br>MDR ARG: AS effluent (35%) and AS influent (50%). ARG for MLS (mefA, mel, ErmB, and mphD) and tetracycline (tetW and tetQ) were dominant in the influent but decreased in the effluent. ARG for aminoglycoside resistance (APH(6)-Id and aadA5), tetracycline (tet44), rifamycin (rpoB), macrolide (mtrA, EreA2), quinolone (QnrS6) were dominant in the AS effluent. ARG types encoding resistance (from influent to (->) effluent): aminocoumarin (2->5%), cephalosporin (0->2%), and rifamycin (17->1%)	Agrawal (2020). Germany and Namibia, here reported for Germany [35]
Locality: not reported. Source: Hospital wastewater, municipal WWTP (influent, effluent), river surface water, rain overflow basins, groundwater	Period: 2 years, not reported. Sampling: 16 sampling sites; clinical wastewater, in- and effluents of WWTP - 24-hour composite samples. Number of samples: 20: 2 - hospital wastewater, 8 - from 4 municipal WWTPs (influent, effluent), 3 - river surface water, 4 - rain overflow basins, 3 - groundwater	*Enterococcus faecium/faecalis*, *Pseudomonas aeruginosa*, *Enterobacteriaceae*, *Staphylococcus aureus*, and CNS. ARGs: blaVIM-1, vanA, ampC, ermB, and mecA	*Enterococci*, *Staphylococci/CNS*, *P. aeruginosa*, *Enterobacteriaceae* and ARGs blaVIM-1, vanA, ampC, ermB were determined in all samples. Only concentrations are reported.	Alexander (2015). Germany [72]
Locality: not reported. Source: 23 conventionally treated WWTP effluents; 3 clusters of catchment areas: communal (n=11), food production/slaughter	Period: Feb-Nov 2018. Sampling: 4 sampling campaigns; grab samples. Number of samples: 92. Number of isolates: not reported	Taxonomic gene markers and ARGs. Studied ARGs: sul1, ermB, blaTEM, tetM, blaCTX-M-15,	99.8–99.9% of the total ARG consisted of ARGs against macrolides (ermB), tetracycline (tetM), Beta-lactame (blaTEM), and sulfonamide (sul1). 0.03–0.17% of the total ARG: beta-lactame ARGs: blaCMY2, blaCTX-M15, blaCTX-M32, and the carbapeneme resistance gene blaOXA48. 0.0001–0.00016% of the total ARG: carbapeneme resistance blaNDM-1, vancomycin	Alexander (2020). Germany [36]

house wastewater (n=4), hospital (n=8)		blaCTX-M-32, blaOXA-48, mecA, vanA, mcr-1, blaNDM-1	resistance vanA, methicilin resistance mecA, colistin resistance mcr-1	
Locality: the Danube River Basin. Germany: Augsburg. Austria: Amstetten. Source: WWTP on the Danube River Basin, mainly industrial wastewater	Period: Aug, Sep 2017. Sampling: WWTP composite effluent samples, 7 liters (1 L for every day for a week), 0.5 L used for analysis of ARGs. Number of samples: 12. Number of isolates: not reported	Quantified ARGs: aph(III)a, blaKPC, blaOXA, blaSHV, ermB, ermF, mecA, qnrS, sul1, tetB, tetM, vanA and vanB	Detected ARGs in Austria and Germany: int1, sul1, ermB, tetM, ermF, blaOXA, aph(III)a (descending oder of concentration). Comparing to other countries, the minimum number of detected ARGs was observed at the WWTPs Amstetten and Augsburg (7 ARGs).	Alygizakis (2019). 9 countries including Germany, Austria [37]
Locality: a nursing home located in south-west Germany. Source: Sewage samples, water samples upstream from municipal sewage from the village and downstream of the nursing home	Period: Nov 2012-Mar 2013. Sampling: Up- and downstream sewage water samples; samples every two hours over a 24-h period, combined as "24-h composite samples". Number of samples: 4	ARGs: metagenomic approach	Detected ARGs: rpoB, tetW, gyrA, aminoglycoside adenyltransferases, macrolide resistance genes ermB and macB, the multidrug gene acrB (resistance-nodulation-division family), quinolone resistance genes parC and both tetracycline genes tetO and tet32. Lower abundance: sulfonamide resistance genes such as folP, sul1, sul2 and vanocmycin resistance related genes such as vanC, vanR, vanS, vanT, vanWvanXYC, vanY and vanZ	Bäumlisberger (2015). Germany [38]
Locality: multiple. Source: wastewater: a hospital-associated wastewater treatment plant in Basel; rural and urban surface water	Period: 2012-2019. Sampling: Wastewater samples collected over 1 year at different influent and effluent sampling sites; screening surface water samples from geographically distributed rivers in Switzerland for fosfomycin-resistant *Enterobacteriaceae*; 100mL water samples. Number of samples: 58 rural and urban water, 128 surface water samples from screening; number of wastewater samples is not given. Number of isolates is reported	*E. coli* and *Klebsiella* spp, fosA-carrying plasmids	Urban/ rural water: 74 isolates ESBL-producing *Enterobacteriaceae* of them 1 fosfomycin-resistant isolate. Wastewater: 59 carbapenem-or amikacin-resistant Enterobacteriaceae isolates, of them 6 fosfomycin-resistant isolates. 3 isolates from the screening.	Biggel (2021). Switzerland [39]

Locality: Area of Graz, Styria. Source: The basin of the incoming untreated wastewater at a sewage treatment plant	Period: Sep 2011-Feb 2012. Sampling: Twice a month; sludge samples were collected using sterile wide-mouth bottles. Number of samples: 11. Number of isolates is reported	ESBL-harbouring *Enterobacteriaceae.* Screening for: blaCTX-M-1group, blaCTX-M-2group, blaCTX-M-9 group, blaGES, blaSHV, blaTEM, and blaVEB<br><br>MRSA, Vancomycin-resistant *Enterococcus* (VRE)	10 (82%) of 11 samples were positive for ESBL-harbouring *Enterobacteriacea*, 3 (27%) samples were positive for MRSA and 4 (36%) samples for VRE.<br>117 *Enterobacteriaceae* isolates, of them MDR: 21 *E. coli*, 7 *Klebsiella pneumoniae*, 3 *Enterobacter* spp. and 1 *Raoultella ornithinolytica*.<br>32 isolates with ESBL genes: 28.6% blaCTX-M-15, 14.3% blaCTX-M-1, 11.9% blaCTX-M-14, 7.1% blaCTX-M-3, 2.4% blaCTX-M-38. Co-resistance: quinolones, nalidixic acid 75% (24 of 32), ciprofloxacin 56.3% (18 of 32) and moxifloxacin 53.1% (17 of 32). Co-resistance for tetracycline was 53.1% (17 of 32) and for trimethoprim/ sulfamethoxazole 50% (16 of 32). Co-resistance rates to aminoglycoside compounds were low with 34.4% (11 of 32) for gentamicin and 0% for amikacin.<br>VRE: *Enterococcus faecium*, harboured the vanA gene. VRE resistant to ampicillin, teicoplanin, and vancomycin.<br>MRSA: one t032, with resistance to erythromycin, norfloxacin and gentamycin and one t067 with resistance to erythromycin, clindamycin and norfloxacin	Galler (2018). Austria [40]
Locality: not reported. Source: 7 WWTPs with different population equivalents and catchment areas	Period: not reported. Sampling: 24 h-composite influent samples 100mL; WWTP effluent 300 mL. Number of samples: not reported. Number of isolates: not reported	Screening for mcr-1 colistin resistance gene	The presence of the colistin resistance gene was documented for all of the influent wastewater samples of the seven WWTPs. The mcr-1 resistance gene was also detected in effluent samples of the WWTPs after conventional treatment. Other 6 quantified ARGs: ermB, tetM, CTX-M-32, blaTEM, CMY-2, and CTX-M	Hembach (2017). Germany [41]
Locality: East Westphalia-Lippe. Source: 4 stages of meat production: slaughterhouses, meat-processing plants, fresh food products and the urban environment	Period: 2018 - 2019. Sampling: From the EU-wide zoonoses-monitoring program. Number of samples: Water: 37, Sewage/Sludge/Soil: 17. Number of isolates is reported	Screened for ESBL-producing, carbapenemase-producing *Enterobacterales* and colistin-resistant *Enterobacterales*	Water: 0 isolates. Sewage/Sludge/Soil: 4 VRE isolates, 8 ESBL-producing E. coli isolates. Antimicrobial resistance genes: 28% in CPE bacteria, 9% in Col-E, 7% in VRE, 15% in ESBL	Klees (2020). Germany [73]
Locality: Clinical/urban wastewater: North-Rhine	Period: Sep 2016-Jul 2017. Sampling: From sewer and	MDR and XDR bacteria	1043 strains: 568 in the rural system (388 of these isolates wastewater or treated wastewater) 475 in the	Müller (2018). Germany [42]

Westphalia. Water from rural area: small river in western part of Germany. Source: Wastewater discharge from hospital buildings; inflow to the receiving municipal sewage treatment plant; WWTP effluent; receiving river upstream and downstream of the WWTP discharge; rural wastewater	WWTP every 6 weeks as automated 24 h mixed samples. Number of samples: 16. Number of isolates: 1043	ACB complex, *E. coli*, *K. pneumoniae/oxytoca*, the *Enterobacter cloacae* complex, and *P. aeruginosa*	clinical/urban system (456 of the isolates were obtained from wastewater or treated wastewater). Clinical/urban: 99.58% cefotaxim/ceftazidim resistant, 25.05% 3MRGN, 28.42% 4MRGN; 9.68% 4MRGN, colistin, positive carbapenemase; 5.47% XDR. Rural: 98.94% cefotaxim/ceftazidim resistant, 15.49% 3MRGN,0.70% 4MRGN; 0.18%% 4MRGN, colistin, positive carbapenemase; 0.00% XDR	
Locality: southern Austria. Source: Sewage, sludge and receiving waters from 3 different sewage treatment plants	Period: Apr-Sep 2000. Sampling: 500 ml sewage, 5 sampling days, sites: inflow (crude sewage), activated sludge tank, effluent, receiving water before outlet (upstream), receiving water after outlet (downstream); 50 g sludge after sewage-sludge press. Number of samples: 5 from each WWTP and site. 767 *E. coli* isolates	*E. coli*	767 *E. coli* isolates: 307 (40%) were resistant against one or several of the 24 antibiotics tested; 244 (31.8%) showed an acquired resistance; out of these 244 isolates, 170 (69.7%) showed a single resistance phenotype, 50 (20.5%) double resistance and 24 (9.8%) - MDR. Penicillin group: highest resistance rates against ampicillin (up to 18%) and piperacillin (up to 12%); in the cephalosporin group: for cefalothin (up to 35%) and cefuroxime-axetil (up to 11%); in the group of quinolones: for nalidixic acid (up to 15%); and for trimethoprime/sulfamethoxazole (up to 13%) and for tetracycline (57%)	Reinthaler (2003). Austria [43]
Locality: district of Kleve. Source: Wastewater, sewage sludge and effluent water; secondary WWTP	Period: May 2018-Apr 2019. Sampling: each week. Number of samples: 36. Number of isolates is not reported	bla and mcr genes in the isolates	244 bla genes of types: blaOXA-58, blaOXA-23, blaOXA-48, blaGES, blaVIM, blaCTX-M-1, blaCTX-M-9, blaCMY-2, blaFOX, blaACT-MIR and blaDHA found in all samples. mcr-1 was detected only in 8 (9%) wastewater samples	Schages (2020). Germany [44]
Locality: the Ruhr Metropolis. Source: community wastewater, no in-patient health care facility in the catchment area; 3 sampling stations in socio-spatially different	Period: Apr 2019-Mar 2020. Sampling: once per month from 3 sampling points and one sampling point at the WWTP. Number of samples: 48. 112 *E. coli* isolates	*E. coli* and ESBL-producing *E. coli*	Phenotypic ESBL-producing *E. coli* were isolated from every sampling point in every month. 112 *E. coli* isolates: no resistance to amikacin, tigecycline or fosfomycin, neither to imipenem or meropenem. 3MRGN resistant to piperacillin, cefotaxime and ciprofloxacin: 19.2% - 31.0% in the areas and 37.0% in WWTP. High prevalence of resistance for piperacillin	Schmiege (2021). Germany [45]

districts; the influent of the local municipal WWTP			(112/112) and cefotaxime (111/112). Ceftazidime: 34.6% - 58.6% in the areas and 55.6% in WWTP; ciprofloxacin: 19.2% - 31.0% and 35.8%; chloramphenicol: 13.8% - 15.4% in areas and 11.8 in WWTP; trimethoprim-sulfamethoxazole: 50.0% - 60% in the areas and 33.3% in WWTP	

Table S5. Presence of antibiotic resistance or antibiotic resistance genes in the environment of animal husbandry as reported in the included studies (n = 8)

Source	Samples	Target	Reported ARB, ARGs presence	Refs
Locality: Nationwide. Source: Piggery manure; manure storage tanks of 15 farms	Period: May - June 2006. Sampling: Manure from storage tanks collected for c. 6 months. Number of samples: 16. Number of isolates: not reported	Plasmids resistant to amoxicillin, sulfadiazine and tetracycline	Among the 81 plasmids, 44 carried the bla-TEM gene, 23 carried sul1, 46 sul2 and 6 sul3. Many plasmids carried bla-TEM and one or two genes coding for sulfadiazine resistance (sul1, sul2 or sul3). 7 plasmids with the sulfadiazine resistance phenotype carried 2 different sul genes	Binh (2008). Germany [46]
Locality: not reported. Source: environmental swabs, manure, flies, mice faeces, farmers dogfaeces, air samples collected with impingers	Period: 2011-2012. Sampling: Retrospective analysis of samples collected in longitudinal studies within the German national ESBL-surveillance project RESET. Number of samples: Environmental swabs (n = 12), manure (n = 9), flies (n = 4), mice faeces (n = 3), farmers dogs faeces (n = 3), air samples collected with impingers (n = 2). Number of isolates is reported	VIM-1-positive *E. coli* and *Salmonella*	40 blaVIM-1-positive isolates total. 22 *E. coli* isolates in single faeces, manure, flies.	Fischer (2017). Germany [47]
Locality: not reported. Source: Boot swabs, flies, dog faeces and manure	Period: 2011 - 2012. Sampling: Samples collected within the German national ESBL-surveillance project RESET. 7 pig farms. Number of samples: Boot swabs (25), barn flies (n = 3), a barn dog (n= 1), manure (n = 5) and mice faeces (n = 1). Number of screened isolates is not reported	mcr-1-positive *E. coli*	7 *mcr-1*-positive *E. coli* strains originating from boot swabs and emission sources such as dog faeces, stable flies and manure.	Guenther (2017). Germany [48]
Locality: Bavaria. Source: Manure samples	Period: Autumn 2002 - Spring 2003. Sampling: at the time of manure application to soil. One manure sample per farm.	*Salmonella spp*	7 samples (1.6%) contained *Salmonella*. 5 out of 7 manure samples (72%) contained antibiotic resistant isolates, all of them were MDR.	Hölzel (2008). Germany [49]

	Number of samples: 380. Number of isolates: not reported			
Locality: not reported. Source: Process water from delivery and unclean areas, wastewater from the in-house WWTP	Period: December 2016 - September 2018. Sampling: Water: 1 Liter; 2 slaughterhouses. 7 sites: water from cleaning transport trucks, transport crates, stunning facilities, scalding water, eviscerators, production facilities, and the influent and effluent. Number of samples: Process water: 50; wastewater: 32. Number of isolates is reported	*Enterococcus spp., Staphylococcus aureus, Klebsiella pneumoniae, Acinetobacter baumannii, Pseudomonas aeruginosa, Enterobacter spp., E. coli*	At least one of the target species was detected in 87.5% of the wastewater samples and 86.0% of the process water samples. Isolates (94.9%, n = 448/472): *E. coli* (39.4%), the A. calcoaceticus-A. baumannii (ACB) complex (32.4%), *S. aureus* (12.3%), and *K. pneumoniae* (10.8%). 58 MRSA isolates. 15-19 E. coli isolates harboring blaCTX-M genes and expressing the 3MDR. Among 93 *E. coli* isolates >90% resistance to piperacillin, cefotaxime, ceftazidime; the most common gene was blaTEM, followed by blaCTX-M and blaSHV. 77 ACB complex from each house: 100% resistance to temocillin, fosfomycin and cefotaxime	Savin (2020). Germany [50]
Locality: North Rhine-Westphalia, Lower Saxony. Source: Dust and manure samples	Period: February 2013 - September 2013. Sampling: 41 pig farms. Number of samples: Samples used for ESBL-producing enterobacteria detection (number not reported) and 6 additional dust and manure samples. 344 CoNS isolates	CoNS, MSSA, MRSA	*S. sciuri* (158/344, 46%), *S. simulans* (50/344, 14%), *S. chromogenes* (34/344, 9.9%), *S. pasteuri* (26/344, 7.6%) and *S. haemolyticus* (21/344, 6.1%). *S. sciuri* was detectable in dust and liquid manure in the majority of the farms (34 of 41; 83%). 65% (184) of the CoNS were resistant to benzylpenicillin and 64% (222) to oxacillin, 38% (130/344) - to erythromycin and 54% (187/344) to clindamycin, 71% (243/344) - to tetracycline, 17% (59/344) - spectinomycin, 8.7% (30/344) chloramphenicol- and 5.5% (19/344) florfenicol-resistant; 20% (70/344) - trimethoprim/sulfamethoxazole, 3.5% - rifampicin (12/344), 19% (66/344) - fosfomycin. *S. sciuri* isolates: 74% (97/131) were resistant to benzylpenicillin, 87% (137/158) - tetracycline, 99% to oxacillin resistant (156/158), of them 67% harboured the mecASCC gene	Schoenfelder (2017). Germany [51]
Locality: Bavaria. Source: Liquid manure samples from pig farms	Period: Autumn 2002 - spring 2003. Sampling: Samples originate from representative monitoring; one sample per farm; at the time of manure application to the fields. Number of samples and isolates: 179.	*Enterococcus*, tetracycline ARGs	Doxycycline resistant *E. faecalis* (n = 147) and phenotypically doxycycline susceptible *E. faecalis* (n = 32). Of 147 doxycycline resistant *E. faecalis*, 127 (86%) carried the tet(M) gene, 94 strains (64%) tet(L), 11 strains (7%) tet(S), and 7 strains (5%) tet(O).	Schwaiger (2009). Germany [52]

Locality: Lower Saxony. Source: Pig manures, 8 pig fattening and 6 pig breeding farms. Digestates from 8 biogas plants	Period: Autumn 2012. Sampling: Samples from cellars, silos, or lagoons using a probe sampler, a bypass sampler or in backflush mode from the vacuum tanker. 4 samples from each farm, 8L. Number of samples: 56	ARGs: sul1, sul2, sul3, tet(A), tet(M), tet(X)	ARGs sul1, sul2, tet(A), tet(M), tet(X), qacEdelta1 were detected in the samples. The occurrence is not quantified.	Wolters (2016). Germany [53]

Table S6. Presence of antibiotic resistance or antibiotic resistance genes in wildlife as reported in the included studies (n = 8)

Source	Samples	Target	Reported ARB, ARGs presence	Refs
Locality: Bavaria, Mecklenburg-Pomerania, Saxony-Anhalt, Saxony and Brandenburg. Source: Wild rodents, feacal samples, rural areas	Period: 2007 - 2008. Sampling: Samples from 8 rodent and 1 shrew species. Sampling sites: agriculturally and silviculturally used farmland and natural preserve areas. Different sampling schemes including rodent monitoring programmes in woodland in Bavaria, Mecklenburg-Pomerania, Saxony-Anhalt, Saxony and Brandenburg and longitudinal studies in Hanta and Dobrava-Belgrad virus endemic areas of the network "Rodent-borne pathogens". Number of samples: 1443. Animals were sampled once, and one colony per sample was further processed. Number of isolates is reported	Targeted ARB: *E. coli*; ARGs: tetracycline (tet(A), tet(B) and tet(C)), sulfonamides (sul1, sul2 and sul3), streptomycin–spectinomycin (aadA, strA and strB), and apramycin–gentamicin (aac(3)-IV)	188 faecal isolates (from 188 samples). 5.5% of the isolates exhibit resistant phenotypes against at least one antimicrobial compound including beta-lactams, tetracyclines, aminoglycosides and sulfonamides. 10 phenotypic resistance isolates were preselected. *E. coli* preselected isolates: 2% of all isolates were resistant against ampicillin, 3% against ticarcillin, 4% against streptomycin, 3% against spectinomycin, 0.5% against sulfamethoxazole/trimethoprim, 0.5% against kanamycin and 1.5% against tetracycline. Resistances to cephalosporins, chloramphenicol and fluoroquinolones were not observed	Guenther (2010). Germany [54]
Locality: Öttingen, Kösingen (Germany); Vienna, Miesenbach, Rohr-Gebirge, Orth an der Donau, Schützen, Klein-Rust, Eggenburg, Zell-See (Austria). Source: Mouflons (Ovis orientalis musimon)	Period: Mar 2012-Jan 2016. Sampling: Nasal and perineal swabs and/or organ samples from free-living 32 mouflons. Hunted game animal species. Dead mouflons submitted explicitly for diagnostic pathological examinations in order to determine their respective cause of death. Sent to Research Institute of Wildlife Ecology for postmortem analysis. Number of samples: 32. Number of isolates is reported	Selection of broad-spectrum cephalosporin-resistant *Enterobacteriaceae* and enterobacterial isolates with reduced susceptibility to fluoroquinolones	1 *E.coli* ESBL phenotype isolate was resistant to all antimicrobial agents tested except imipenem and colistin: ampicillin, amoxicillin/clavulanic acid, ceftiofur, cefquinome, cephalotin, cefotaxime, cefoperazone, nalidixic acid, streptomycin, neomycin, gentamicin, ciprofloxacin, enrofloxacin, trimethoprim-sulfamethoxazole, tetracycline, doxycycline, chloramphenicol, florfenicol.	Loncaric (2016). Austria, Germany [55]
Locality: Migratory population in the Lobau. Non-migratory	Period: Mar 2013. Sampling: Fresh faeces. Number of samples: 54 from migratory and 102 from a resident	beta-lactamase producing *Enterobacteriaceae* and methicillin-	24 cefotaxime-resistant *Enterobactericeae* isolates were obtained from the migratory population; 3 cefotaxime-resistant enterobacterial isolates were obtained from the resident population. 22 isolates displayed ESBL	Loncaric (2013). Austria [56]

population in Wulkaprodersdorf. Source: Rooks (Corvus frugilegus) 2 populations: migratory and a resident population.	population. Number of isolates is reported	resistant *Staphylococcus aureus*. Isolation of cefotaxime resistant isolates.	phenotype, and 5 isolates displayed AmpC phenotype, wherefrom 3 *Enterobacter* isolates were stably depressed AmpC-producer. The most prevalent genes in ESBL-producing isolates were from the blaCTX-M group. 5 isolates of MRSA: detected in 5 samples of the migratory population; resident population - none. In all 5 isolates the production of beta-lactamase was confirmed. All 5 isolates were mecA and PVL positive. 44.4% of the faecal samples (24 of 54) in the migrating population showed at least resistance to one of the tested antibiotics compared to 2.9% (3 of 102) in the resident population. Migratory population: detected resistance to amipicillin and piperacillin antibiotics, tetracycline (85% of the isolates), trimethoprim/ sulfamethoxazole (62%) and chloramphenicol (48%). Resident population: 3 cefotaxime-resistant isolates showed phenotypic resistance to amipicillin, piperacillin, and tobramycin.	
Locality: Nationwide. Source: Wild boars (Sus scrofa), roe deer (Capreolus capreolus), wild ducks (family Anatidae, subfamily Anatinae) and geese (family Anatidae, subfamily Anserinae)	Period: 2016 (wild boar), 2017 (roe deer), 2019 (wild ducks and geese). Sampling: National Zoonoses Monitoring Program. Faecal samples. Nasal swabs from wild boars. Tested in the National Reference Laboratories. Samples from wild ducks and geese originated from cadavers collected for the monitoring of avian influenza, or taken from hunted birds. Number of samples: 942 samples from hunted wild boars, 573 from roe deer and 100 from wild ducks and geese.	*Salmonella spp.* (in wild boars and wild ducks and geese), *Campylobacter spp*. (in roe deer and wild ducks and geese), Shiga toxin-producing *E. coli* (STEC), commensal *E. coli* and extended-spectrum beta-lactamase- (ESBL) or ampicillinase class C (AmpC) beta-lactamase-producing *E. coli*. MRSA in boars. Resistance test was not carried out in all isolates.	*Salmonella spp.:* isolated from wild boar (13 isoaltes, 2.4% samples), wild ducks and geese feacal samples. *Campylobacter spp*.: roe deer (4 samples, 0.8%), wild ducks and geese feacal samples. STEC: wild boar (37, 6.9%), roe deer (144, 40.2%) feacal samples. MRSA: wild boar nasal swabs (5 samples). Commensal *E. coli*: wild boar (511 samples, 95%), roe deer (537, 93.7%), wild ducks and geese (51, 50.0%) feacal samples. ESBL-/AmpC-producing E. coli: wild boar (36, 6.5%), roe deer (13, 2.3%), wild ducks and geese (10, 9.8%) feacal samples.<br>Wild boars*: Salmonella spp.* - chloramphenicol , nalidixic acid, ciprofloxacin, ampicillin, colistin, tetracycline - 9.1%, 2x resistant and 3x resistant- 9.1%; STEC - gentamicin (4.2%), chloramphenicol (4.2%), nalidixic acid (4.2%), ciprofloxacin (4.2%), ampicillin (4.2%), sulfamethoxazole (4.2%), trimethoprim (4.2%), tetracycline (4.2%), >4x resistant (4.2%) ; Commensal *E. coli* <1% resistant; ESBL-/AmpC- producing *E. coli* -	Plaza-Rodríguez (2020). Germany [57]

			gentamicin (20%), chloramphenicol (16%), cefotaxime (100%), ceftazidime (96%), nalidixic acid (16%), ciprofloxacin (32%), ampicillin (100%), sulfamethoxazole (28%), trimethoprim (24%), tetracycline (36%), azithromycin (8%), 44% 2x resistant, 20% 3x resistant, 32% >4x resistant.<br>Roe deer: STEC - gentamicin (0.8%); commensal *E. coli* - cefotaxime (0.4%), ceftazidime (0.4%), ciprofloxacin (0.4%), ampicillin (1.5%), sulfamethoxazole (1.1%), trimethoprim (0.7%), tetracycline (0.7%), azithromycin (0.4%), >4x resistant (1 isolate, 0.4%); ESBL-/AmpC-producing *E. coli* - gentamicin (8.3%), cefotaxime (100%), ceftazidime (100%), ciprofloxacin (8.3%), ampicillin (100%), sulfamethoxazole (16.7%), trimethoprim (16.7%), tetracycline (8.3%), 2x resistant (75%), 3x resistant (16.7%), >4x resistant (8.3%)<br>Wild ducks and geese: Commensal *E. coli* - gentamicin (2%), cefotaxime (2%), ceftazidime (2%), nalidixic acid (2%), ciprofloxacin (2%), ampicillin (10.2%), colistin (2%), sulfamethoxazole (6.1%), trimethoprim (2%), tetracycline (2%), 2x resistant (2%), 3x resistant (4.1); ESBL-/AmpC- producing *E. coli* - gentamicin (20%), cefotaxime (100%), ceftazidime (100%), nalidixic acid (50%), ciprofloxacin (50%), ampicillin (100%), sulfamethoxazole (60%), trimethoprim (20%), tetracycline (20%), azithromycin (10%), 2x resistant (30%), 3x resistant (20%), 4x resistant (10%), >4x resistant (40%)	
Locality: Brandenburg, Mecklenburg-Western Pomerania. Source: Wild boars	Period: February 2015 - December 2016. Sampling: Tonsils of hunted wild boars. Number of samples: 503. Number of isolates: 10	*Yersinia pseudotuberculosis*	32 samples (6.4%): *Y. pseudotuberculosis* was detected. The isolates were susceptible to most antimicrobials. 4 isolates exhibited growth at trimethoprim; all strains grew at high concentrations of colistin-sulfate	Reinhardt (2018). Germany [58]
Locality: Berlin. Source: Wild birds	Period: 2011 - 2014. Sampling: Rescued wild birds, the small animal clinic, 40 different avian species. Sampled once, the cloacal swab.	ESBL-producing *E. coli*	24 (7.5% of samples) isolates of ESBL/AmpC-producing *E. coli*. Dominance of blaCTX-M-1 (60%) and blaCTX-M-15 (40%) for phenotypic ESBL-producers. Common ARGs: blaTEM-1, blaOXA-1,	Schaufler (2016). Germany [59]

	Number of samples: 320. 24 ESBL-producing *E. coli* isolates.		tetA/B, sul1, sul2, sul3, strA/strB. 14 of 24 isolates (4% of samples) were MDR.	
Locality: Berlin, urban centre. Source: Flies	Period: July 2016. Sampling: Sampling sites: 4 sites within a quadrant of 5 km in the city centre of Berlin. Flies: blowflies (*Diptera Calliphoridae*), houseflies (*Diptera Muscidae*), or flesh flies (*Diptera Sarcophagidae*). Number of samples: 163. Number of isolates: 24	ESBL-producing *E. coli*	21 (12.9%) samples were positive for ESBL-producing *E. coli.* 10 samples with ESBL-producing *E. coli* (6%) showed a co-resistance to fluoroquinolones (ciprofloxacin). 5 ESBL-positive fly samples (3%) showed additional non-susceptibility to the group of folic acid antagonists (trimethoprim/sulfamethoxazole). No resistances to carbapenems or colistin were detected. ARG CTX-M-1 was the most prevalent type 15 (of 24)	Wetzker (2019). Germany [60]
Locality: the Swiss Ornithological Institute in Sempach. Source: Wild birds admitted to the care centre, the rehabilitation centre	Period: May - October 2018. Sampling: From live birds, swabs of freshly passed faeces, from dead birds a faecal swab from the cloaca; 55 different species. The majority (246/83.7%) were nestlings, pulli, or juvenile birds. Number of samples: 294 faecal swabs from 294 wild birds. 256 E. coli isolates	antimicrobial resistant *E. coli*	17 samples (5.8%) contained 19 AMR *E. coli* isolates, where of 26.3% were MDR. Five (1.7%) ESBL-producing *E. coli*. Within AMR *E. coli*, high rates of resistance to ampicillin (8/19 isolates), nalidixic acid (8/19), and tetracycline (10/19) were observed. Resistance to chloramphenicol (5/19), ciprofloxacin (4/19), cefazolin (4/19), streptomycin (5/19), and sulfamethoxazole/trimethoprim (5/19) were less often detected, and resistance to amoxicillin/clavulanic acid (1/19), azithromycin (2/19), nitrofurantoin (1/19), gentamicin (2/19), and kanamycin (2/19)	Zurfluh (2019). Switzerland [61]

Table S7. Presence of antibiotic resistance or antibiotic resistance genes in sediment and soil as reported in the included studies (n = 10)

Source	Samples	Target	Reported ARB, ARGs presence	Refs
Locality: Baltic Sea and North Sea coastline (environmental isolates), bathing sites. Source: Coastal water, sediment, bivalve mollusks	Period: 2004–2012, 2009-2014. Sampling: not reported. Number of samples (isolates): *V. cholerae*: 131; *V. vulnificu*: 122. Isolates: 141 *V. vulnificus*; 184 *V. choleraenon*-O1/non-O139	*V. cholerae* non-O1/non-O139; *V. vulnificu*	15% showed full resistance to at least one antimicrobial agent. None of the *V. cholerae* isolates showed multidrug resistance. Non-susceptible strains: 34% in *V. cholerae*; 41% in *V. vulnificu*. *V. cholerae*: amoxicillin/clavulanic acid 0; ampicillin 10 (7.6%); imipenem 3 (2.3%); meropenem 1; nalidixic acid 0; streptomycin 2 (1.5%); trimethoprim 0. *V. vulnificu*: streptomycin 2%; ampicillin 11%	Bier (2015). Germany [62]
Locality: Vidy Bay, Lake Geneva. Source: Freshwater lake sediments	Period: Aug 2011. Sampling: Short sediment cores taken at 22 sites during two sampling campaigns. Number of samples: not reported	ARGs: sul1, sul2, tet(B), tet(M), tet(W) and qnrA	ARG abundance generally decreased from sul1 over sul2, tet(w), tet(M) to tet(B); qnrA was detected in low amounts and only in hospital sewage; sul and tet were more abundant in both WWTP influent and effluent samples, with sul1 abundance always being the highest. For all the detected ARGs, highest gene concentrations and abundances occurred at sites in close proximity to the WWTP discharge	Czekalski (2014). Switzerland [64]
Locality: Vidy Bay (Lake Geneva), Lausanne. Source: Hospital and municipal raw sewage, treated effluent from Lausanne's WWTP, lake water, sediment samples close to WWTP and close to a drinking water pump	Period: not reported. Sampling: 3 sampling campaigns. 1 L wastewater, lake water, sediment. Number of samples: not reported. 163 isolates from wastewater.	-	Clinical wastewater: *Enterococci and Brevundimonas* were dominant; *Escherichia/Shigella* and Acidovorax species dominated the WWTP isolates. 17 genera were identified among the lake water bacteria. Sediment: *Bacillus, Solibacillus, Brevundimonas* were frequent. 76% of hospital and 77% of WWTP isolates resistant to sulfamethoxazole/trimethoprim and streptomycin were additionally resistant to ampicillin. WWTP: 55% resistant to chloramphenicol, 67% resistant to nalidixic acid. MDR to 6-8 antibiotics: 86 and 77% in hospital wastewater, 82 - 60% wastewater. MDR *Pseudomonas* and *Escherichia/Shigella* were present at all 3 locations	Czekalski (2012). Switzerland [63]
Locality: Vidy Bay, Lake Geneva. Source: Sediments from the	Period: May 2012. Sampling: 3 sites; 35-60m depth, surface sediment. Number of samples: not reported. 141 isolates	*Pseudomonas spp: P. putida, P. aeruginosa*	12 - 54% of antibiotic resistance among the isolates. MDR isolates were identified in all 3 sampling areas; 2 isolates were resistant to 16 antibiotics. Beta-lactamases in the studied *Pseudomonas spp*: ctx-m 47%, vim-2 19%, vim-1 22%, ndm 6%, shv - 6%. 38 and 58% of the isolates were resistant to streptomycin and cefoperazone, respectively; 54% to ofloxacine; 22% -	Devarajan (2017). Switzerland [65]

			norfloxacine; <20%: imipenem, ceftazidime, piperacillin-tazobactam, cefepime, piperacillin	
Locality: the Lake Brêt. Source: Sediments from the Lake Brêt used as drinking-water reservoir	Period: Sep 2017. Sampling: Sites: in the middle of the lake, 3m from the shore, on the water line, and input location in the lake of an agricultural drain. Number of samples: not reported	ARGs: blaTEM, blaSHV, blaCTX-M and blaNDM	blaTEM and blaSHV were found in all sampling sites; blaCTX-M—only in the samples from the shore; blaNDM gene was not identified in sediments; high abundance of blaTEM and blaSHV were observed in the center of the lake; sul1 and sul2 were detected in all the studied sites. On agricultural soil, blaCTX-M, blaNDM, and sul1 were not quantifiable	Laffite (2020). Switzerland [66]
Locality: Rax, Regelsbrunn. Source: Soil and water	Period: 2007 - 2009. Sampling: 12 sampling areas, samples of soil with different compositions at different altitudes (0 to 500 m, 500 to 1,000 m, and >1,500 m). Number of samples: 467 soil and 68 water samples	*Listeria* species	30% (n = 140) were determined to be positive for *Listeria spp*., of which 28 samples (6%) were *L. monocytogenes* positive. *Listeria* was isolated from 26.5% of water samples. 26 *L. monocytogenes* isolates were tested for resistance. *L. monocytogenes* strains: 88.46% resistant against cefotaxime, 65.4% - erythromycin, 35%, 12%, and 6% of *L. monocytogenes* test strains were resistant to ceftriaxone, ciprofloxacin, and linezolid	Linke (2014). Austria [69]
Locality: The urban park Berlin Tiergarten and the abandoned sewage field Berlin-Buch. Source: Soil under anthropogenic influences and soil which used to be under wastewater application	Period: not reported. Sampling: From each site 1000 g of soil sample was collected, depth 3- 15 cm. Number of samples: 6	ampC, tet (O), ermB, SHV-5, mecA, and vanA	ARGs were not quantified in the German soil	Malik (2008). Germany, India[70]
Locality: The Kraichbach River, Baden-Württemberg. Source: WWTP-impacted river in surface water, sediment, and biofilm. The river is	Period: February - June 2019. Sampling: Surface water - grab sampling, sediment - core sampling (5 cm), and biofilm - passive sampling. 5 sampling campaigns. Number of samples: 20, details are not reported. Number of isolates reported	blaTEM, ermB, tetM, and sul1 in all samples. Not all of the samples and isolates were analyzed for resistance	Two sediment samples and two biofilm samples (one upstream and one downstream) were tested for antibiotic resistance. The selected isolates from sediment: *Klebsiella, Enterobacter, Citrobacter* (3 isolates), *E. coli* (3 isolates), and *Acinetobacter*. Many isolates were resistant to cephalosporins (either cefotaxime or ceftazidime, or both). No isolate collected upstream of the WWTP was classified as 3MRGN or 4MRGN. Downstream of the WWTP, the isolates belonged to the species *P. aeruginosa* (5 isolates) and *Acinetobacter* (5 isolates). *E. coli* was the only species successfully isolated from the biofilm	Reichert (2021). Germany [67]

the effluent receptor of 6 WWTPs				
Locality: Lake Geneva: The Bay of Vidy and the Creux de Genthod region. Source: Sediment from 2 sites: close to the outlet pipes of WWTP and coastal area	Period: not reported. Sampling: Sediment cores at different depths. Number of samples: not reported	*E. coli* and *Enterococcus*; ARGs: beta-lactams, aadA, tet, cmlA, van	*E. coli* MDR: 0.12% - 4.6%; *Enterococcus* MDR: 0.016% - 11.6%. *E. coli* beta-lactames resistant: 21.94 - 48.22%; *Enterococcus* beta-lactames resistant: 15.92 - 36.82%. aadA found in all the studied samples	Thevenon (2012). Switzerland [68]
Locality: Maize and potato growing regions. Source: Agricultural soils: 50 maize and 50 potato fields not yet exposed to but eligible for GMO crop cultivation	Period: Aug-Sep 2011. Sampling: 10 single soil extractions from each field; extraction layer: 0-25 cm. Number of samples: 100	nptII, nptIII (present in transgenic plants)	85 (85%) soil samples were positive for nptIII and 6 (6%) for nptII. Potato fields: nptII - 6%; nptIII - 78%. Maize: nptII - 6%; nptIII -92%. 400 kanamycin-resistant strains. Strains: Maize - taxons of *Sphingobacteriaceae (Pedobacter sp.), Flavobacteriaceae (Chryseobacterium sp.), Rhizobiaceae (Ensifer sp.)* and *Xanthomonadaceae (Stenotrophomonas); Potato - Rhizobiaceae (Ensifer adhaerens), Sphingobacteriaceae (Pedobacter sp.), Flavobacteriaceae, and Chitinophagaceae*. 3 maize and 3 potato fields carried bacterial strains known as human pathogens: *Klebsiella pneumoniae, Stenotrophomonas maltophilia*. Kanamycin resistance: Maize - mean 6.22%, range 0.34 - 16%; Potato: mean 5.42%, range 0.14 - 10.94%	Woegerbauer (2015). Austria [71]

Table S8. Reported resistance patterns categorized by associated antibiotic class, WHO CIA list rank and environmental reservoir.

Environment	WHO CIA list category	Antibiotic Class	Reported resistance	Samples
Animal husbandry	Critically important: Highest Priority	Polymyxins	mcr-1	Farm sites [Germany, 2011-2012 (Guenther)]
Animal husbandry	Critically important: Highest Priority	Third-generation cephalosporins	cefotaxime	Wastewater [Germany, Dec2016-Sep2018 (Savin)]
Animal husbandry	Critically important: Highest Priority	Third-generation cephalosporins	ceftazidime	Wastewater [Germany, Dec2016-Sep2018 (Savin)]
Animal husbandry	Critically important: High Priority	Carboxypenicillins	temocillin	Wastewater [Germany, Dec2016-Sep2018 (Savin)]
Animal husbandry	Critically important: High Priority	Macrolides	erythromycin	Dust,manure [Germany, Feb2013-Sep2013 (Schoenfelder)]
Animal husbandry	Critically important: High Priority	Phosphonics	fosfomycin	Wastewater [Germany, Dec2016-Sep2018 (Savin)];Dust,manure [Germany, Feb2013-Sep2013 (Schoenfelder)]
Animal husbandry	Critically important: High Priority	Rifamycins	rifampicin	Dust,manure [Germany, Feb2013-Sep2013 (Schoenfelder)]
Animal husbandry	Highly important	Amphenicols	chloramphenicol	Dust,manure [Germany, Feb2013-Sep2013 (Schoenfelder)]
Animal husbandry	Highly important	Amphenicols	florfenicol	Dust,manure [Germany, Feb2013-Sep2013 (Schoenfelder)]
Animal husbandry	Highly important	Lincosamides	clindamycin	Dust,manure [Germany, Feb2013-Sep2013 (Schoenfelder)]
Animal husbandry	Highly important	Penicillins	benzylpenicillin	Dust,manure [Germany, Feb2013-Sep2013 (Schoenfelder)]
Animal husbandry	Highly important	Penicillins	oxacillin	Dust,manure [Germany, Feb2013-Sep2013 (Schoenfelder)]
Animal husbandry	Highly important	Penicillins	piperacillin	Wastewater [Germany, Dec2016-Sep2018 (Savin)]
Animal husbandry	Highly important	Sulfonamides	sul1	Piggery manure [Germany, May-Jun2006 (Binh)];Piggery manure [Germany, 2012 (Wolters)]
Animal husbandry	Highly important	Sulfonamides	sul2	Piggery manure [Germany, May-Jun2006 (Binh)];Piggery manure [Germany, 2012 (Wolters)]
Animal husbandry	Highly important	Sulfonamides	sul3	Piggery manure [Germany, May-Jun2006 (Binh)];Piggery manure [Germany, 2012 (Wolters)]
Animal husbandry	Highly important	Tetracyclines	doxycycline	Piggery manure [Germany, 2002-2003 (Schwaiger)]
Animal husbandry	Highly important	Tetracyclines	tetracycline	Dust,manure [Germany, Feb2013-Sep2013 (Schoenfelder)]

Animal husbandry	Highly important	Trimethoprim - sulfonamide combinations	trimethoprim /sulfamethox azole	Dust,manure [Germany, Feb2013-Sep2013 (Schoenfelder)]
Animal husbandry	Important	Aminocyclitols	spectinomyci n	Dust,manure [Germany, Feb2013-Sep2013 (Schoenfelder)]
Animal husbandry	not listed	Antiseptics	qacEdelta1	Piggery manure [Germany, 2012 (Wolters)]
Animal husbandry	not listed	Beta lactamases	blaCTX-M	Wastewater [Germany, Dec2016-Sep2018 (Savin)]
Animal husbandry	not listed	Beta lactamases	blaSHV	Wastewater [Germany, Dec2016-Sep2018 (Savin)]
Animal husbandry	not listed	Beta lactamases	blaTEM	Piggery manure [Germany, May-Jun2006 (Binh)];Wastewater [Germany, Dec2016-Sep2018 (Savin)]
Animal husbandry	not listed	Beta lactamases	blaVIM	Farm sites [Germany, 2011–2012 (Fischer)]
Animal husbandry	not listed	Beta lactamases	blaVIM-1	Farm sites [Germany, 2011–2012 (Fischer)]
Wastewater	Critically important: Highest Priority	Fluoroquinolon es	ciprofloxacin	Wastewater [Austria, Sep2011-Feb2012 (Galler)];Wastewater [Germany, Apr2019-Mar2020 (Schmiege)]
Wastewater	Critically important: Highest Priority	Fluoroquinolon es	moxifloxacin	Wastewater [Austria, Sep2011-Feb2012 (Galler)]
Wastewater	Critically important: Highest Priority	Fluoroquinolon es	norfloxacin	Wastewater [Austria, Sep2011-Feb2012 (Galler)]
Wastewater	Critically important: Highest Priority	Fluoroquinolon es	ofloxacin	Wastewater [Austria, Sep2011-Feb2012 (Galler)];Wastewater [Germany, Apr2019-Mar2020 (Schmiege)]
Wastewater	Critically important: Highest Priority	Glycopeptides	teicoplanin	Wastewater [Austria, Sep2011-Feb2012 (Galler)]
Wastewater	Critically important: Highest Priority	Glycopeptides	vanA	Hospital, municipal wastewater [Germany, 2years (Alexander)]
Wastewater	Critically important: Highest Priority	Glycopeptides	vancomycin	Wastewater [Germany, Feb-Nov2018 (Alexander)];Wastewater(nursing home) [Germany, Nov2012-Mar2013 (Bäumlisberger)];Wastewater [Austria, Sep2011-Feb2012 (Galler)];Wastewater, surface water [Germany, 2018-2019 (Klees)]
Wastewater	Critically important: Highest Priority	Polymyxins	colistin	Wastewater [Germany, Feb-Nov2018 (Alexander)];WWPT [Germany, not reported (Hembach)];Clinical,urban wastewater [Germany, Sep2016-

				Jul2017 (Müller)];Rural wastewater [Germany, Sep2016-Jul2017 (Müller)]
Wastewater	Critically important: Highest Priority	Polymyxins	mcr-1	WWPT [Germany, May2018-Apr2019 (Schages)]
Wastewater	Critically important: Highest Priority	Quinolones	nalidixic acid	Wastewater [Austria, Sep2011-Feb2012 (Galler)];Wastewater [Austria, Apr-Sep2000 (Reinthaler)]
Wastewater	Critically important: Highest Priority	Quinolones	quinolones	WWPT effluent [Germany and Namibia, here report for Germany, not reported (Agrawal)];WWPT influent [Germany and Namibia, here report for Germany, not reported (Agrawal)];Wastewater(nursing home) [Germany, Nov2012-Mar2013 (Bäumlisberger)];Wastewater [Austria, Sep2011-Feb2012 (Galler)]
Wastewater	Critically important: Highest Priority	Third-generation cephalosporins	cefotaxime	Clinical,urban wastewater [Germany, Sep2016-Jul2017 (Müller)];Rural wastewater [Germany, Sep2016-Jul2017 (Müller)];Wastewater [Germany, Apr2019-Mar2020 (Schmiege)]
Wastewater	Critically important: Highest Priority	Third-generation cephalosporins	ceftazidime	Clinical,urban wastewater [Germany, Sep2016-Jul2017 (Müller)];Rural wastewater [Germany, Sep2016-Jul2017 (Müller)];Wastewater [Germany, Apr2019-Mar2020 (Schmiege)]
Wastewater	Critically important: High Priority	Aminoglycosides	amikacin	WWPT [Switzerland, 2012-2019 (Biggel)]
Wastewater	Critically important: High Priority	Aminoglycosides	aminoglycoside	WWPT effluent [Germany and Namibia, here report for Germany, not reported (Agrawal)];WWPT influent [Germany and Namibia, here report for Germany, not reported (Agrawal)];Wastewater(nursing home) [Germany, Nov2012-Mar2013 (Bäumlisberger)];Wastewater [Austria, Sep2011-Feb2012 (Galler)]
Wastewater	Critically important: High Priority	Aminoglycosides	aph3a	Wastewater(Danube River) [9 countries including Germany and Austria, Aug.Sep2017 (Alygizakis)]
Wastewater	Critically important: High Priority	Aminoglycosides	gentamicin	Wastewater [Austria, Sep2011-Feb2012 (Galler)]
Wastewater	Critically important: High Priority	Carbapenems	carbapenem	Wastewater [Germany, Feb-Nov2018 (Alexander)];WWPT [Switzerland, 2012-2019

				(Biggel)];Clinical,urban wastewater [Germany, Sep2016-Jul2017 (Müller)];Rural wastewater [Germany, Sep2016-Jul2017 (Müller)]
Wastewater	Critically important: High Priority	Macrolide–lincosamide–streptogramin	macrolide–lincosamide–streptogramin	WWPT effluent [Germany and Namibia, here report for Germany, not reported (Agrawal)];WWPT influent [Germany and Namibia, here report for Germany, not reported (Agrawal)]
Wastewater	Critically important: High Priority	Macrolides	ermB	Wastewater(Danube River) [9 countries including Germany and Austria, Aug.Sep2017 (Alygizakis)];WWPT [Germany, not reported (Hembach)];Hospital, municipal wastewater [Germany, 2years (Alexander)]
Wastewater	Critically important: High Priority	Macrolides	erythromycin	Wastewater [Austria, Sep2011-Feb2012 (Galler)]
Wastewater	Critically important: High Priority	Macrolides	macrolide	WWPT effluent [Germany and Namibia, here report for Germany, not reported (Agrawal)];WWPT influent [Germany and Namibia, here report for Germany, not reported (Agrawal)];Wastewater [Germany, Feb-Nov2018 (Alexander)];Wastewater(nursing home) [Germany, Nov2012-Mar2013 (Bäumlisberger)]
Wastewater	Critically important: High Priority	Phosphonics	fosfomycin	WWPT [Switzerland, 2012-2019 (Biggel)]
Wastewater	Critically important: High Priority	Rifamycins	rifamycin	WWPT effluent [Germany and Namibia, here report for Germany, not reported (Agrawal)];WWPT influent [Germany and Namibia, here report for Germany, not reported (Agrawal)]
Wastewater	Highly important	Amphenicols	chloramphenicol	Wastewater [Germany, Apr2019-Mar2020 (Schmiege)]
Wastewater	Highly important	Cephalosporins	cephalosporin	WWPT effluent [Germany and Namibia, here report for Germany, not reported (Agrawal)]
Wastewater	Highly important	First-generation cephalosporins	cefalotin	Wastewater [Austria, Apr-Sep2000 (Reinthaler)]
Wastewater	Highly important	Lincosamides	clindamycin	Wastewater [Austria, Sep2011-Feb2012 (Galler)]
Wastewater	Highly important	Penicillins	ampicillin	Wastewater [Austria, Sep2011-Feb2012 (Galler)];Wastewater [Austria, Apr-Sep2000 (Reinthaler)]
Wastewater	Highly important	Penicillins	piperacillin	Wastewater [Austria, Apr-Sep2000 (Reinthaler)];Wastewater [Germany, Apr2019-Mar2020 (Schmiege)]

Wastewater	Highly important	Second-generation cephalosporins	cefuroxime	Wastewater [Austria, Apr-Sep2000 (Reinthaler)]
Wastewater	Highly important	Streptogramins	ermF	Wastewater(Danube River) [9 countries including Germany and Austria, Aug.Sep2017 (Alygizakis)]
Wastewater	Highly important	Sulfonamides	sul1	Wastewater(Danube River) [9 countries including Germany and Austria, Aug.Sep2017 (Alygizakis)]
Wastewater	Highly important	Sulfonamides	sulfonamide	Wastewater [Germany, Feb-Nov2018 (Alexander)];Wastewater(nursing home) [Germany, Nov2012-Mar2013 (Bäumlisberger)]
Wastewater	Highly important	Tetracyclines	tetracycline	WWPT effluent [Germany and Namibia, here report for Germany, not reported (Agrawal)];WWPT influent [Germany and Namibia, here report for Germany, not reported (Agrawal)];Wastewater [Germany, Feb-Nov2018 (Alexander)];Wastewater(nursing home) [Germany, Nov2012-Mar2013 (Bäumlisberger)];Wastewater [Austria, Sep2011-Feb2012 (Galler)];Wastewater [Austria, Apr-Sep2000 (Reinthaler)]
Wastewater	Highly important	Trimethoprim - sulfonamide combinations	trimethoprim /sulfamethox azole	Wastewater [Austria, Sep2011-Feb2012 (Galler)];Wastewater [Austria, Apr-Sep2000 (Reinthaler)];Wastewater [Germany, Apr2019-Mar2020 (Schmiege)]
Wastewater	not listed	Beta lactamases	ampC	Hospital, municipal wastewater [Germany, 2years (Alexander)]
Wastewater	not listed	Beta lactamases	beta-lactames	Wastewater [Germany, Feb-Nov2018 (Alexander)]
Wastewater	not listed	Beta lactamases	blaACT-MIR	WWPT [Germany, May2018-Apr2019 (Schages)]
Wastewater	not listed	Beta lactamases	blaCMY-2	WWPT [Germany, not reported (Hembach)];WWPT [Germany, May2018-Apr2019 (Schages)]
Wastewater	not listed	Beta lactamases	blaCTX-M	WWPT [Germany, not reported (Hembach)];WWPT [Germany, May2018-Apr2019 (Schages)]
Wastewater	not listed	Beta lactamases	blaCTX-M-1	WWPT [Germany, May2018-Apr2019 (Schages)]
Wastewater	not listed	Beta lactamases	blaCTX-M-32	WWPT [Germany, not reported (Hembach)]
Wastewater	not listed	Beta lactamases	blaCTX-M-9	WWPT [Germany, May2018-Apr2019 (Schages)]
Wastewater	not listed	Beta lactamases	blaDHA	WWPT [Germany, May2018-Apr2019 (Schages)]
Wastewater	not listed	Beta lactamases	blaFOX	WWPT [Germany, May2018-Apr2019 (Schages)]

Wastewater	not listed	Beta lactamases	blaGES	WWPT [Germany, May2018-Apr2019 (Schages)]
Wastewater	not listed	Beta lactamases	blaOXA	Wastewater(Danube River) [9 countries including Germany and Austria, Aug.Sep2017 (Alygizakis)];WWPT [Germany, May2018-Apr2019 (Schages)]
Wastewater	not listed	Beta lactamases	blaOXA-23	WWPT [Germany, May2018-Apr2019 (Schages)]
Wastewater	not listed	Beta lactamases	blaOXA-48	WWPT [Germany, May2018-Apr2019 (Schages)]
Wastewater	not listed	Beta lactamases	blaOXA-58	WWPT [Germany, May2018-Apr2019 (Schages)]
Wastewater	not listed	Beta lactamases	blaTEM	WWPT [Germany, not reported (Hembach)]
Wastewater	not listed	Beta lactamases	blaVIM	WWPT [Germany, May2018-Apr2019 (Schages)];Hospital, municipal wastewater [Germany, 2years (Alexander)]
Wastewater	not listed	Beta lactamases	blaVIM-1	Hospital, municipal wastewater [Germany, 2years (Alexander)]
Wastewater	not listed	Beta lactamases	intl1	Wastewater(Danube River) [9 countries including Germany and Austria, Aug.Sep2017 (Alygizakis)]
Wastewater	not listed	Beta lactamases	methicilin	Wastewater [Germany, Feb-Nov2018 (Alexander)]
Wastewater	not listed	Extended spectrum beta lactamases	ESBL	Wastewater [Austria, Sep2011-Feb2012 (Galler)];Wastewater, surface water [Germany, 2018-2019 (Klees)]
Wastewater	Currently not used in humans	Aminocoumarins	aminocoumarin	WWPT effluent [Germany and Namibia, here report for Germany, not reported (Agrawal)];WWPT influent [Germany and Namibia, here report for Germany, not reported (Agrawal)]
Water body	Critically important: Highest Priority	Fluoroquinolones	ciprofloxacin	Danube, Glan, Inn, Traun [Austria, 2016 (Lepuschitz)];Drinking water [Germany, Aug2018-Jun2019 (Voigt)]
Water body	Critically important: Highest Priority	Fluoroquinolones	fluoroquinolones	River Weser [Germany, Jul-Sep2018 (Song)];River Mur [Austria, Oct2015-Jan2016 (Zarfel)]
Water body	Critically important: Highest Priority	Fluoroquinolones	moxifloxacin	Danube, Glan, Inn, Traun [Austria, 2016 (Lepuschitz)]
Water body	Critically important: Highest Priority	Fluoroquinolones	ofloxacin	River Swist [Germany, not reported (Schreiber)];Danube, Glan, Inn, Traun [Austria, 2016 (Lepuschitz)];Drinking water [Germany, Aug2018-Jun2019 (Voigt)]
Water body	Critically important:	Fourth-generation cephalosporins	cefepime	Rivers, inland canals, streams [Switzerland, May-Aug2019 (Bleichenbacher)]

	Highest Priority			
Water body	Critically important: Highest Priority	Polymyxins	colistin	Bathing waters [Germany, May-Sep2018 (Döhla)]
Water body	Critically important: Highest Priority	Quinolones	nalidixic acid	River Mur [Austria, Oct2015-Jan2016 (Zarfel)]
Water body	Critically important: Highest Priority	Quinolones	quinolones	Lakes water [Switzerland, 2002-2005 (Picão)];River Weser [Germany, Jul-Sep2018 (Song)];River Mur [Austria, Oct2015-Jan2016 (Zarfel)]
Water body	Critically important: Highest Priority	Third-generation cephalosporins	cefotaxime	Rivers, inland canals, streams [Switzerland, May-Aug2019 (Bleichenbacher)];Drinking water [Germany, Aug2018-Jun2019 (Voigt)]
Water body	Critically important: Highest Priority	Third-generation cephalosporins	ceftazidime	River Swist [Germany, not reported (Schreiber)];Drinking water [Germany, Aug2018-Jun2019 (Voigt)]
Water body	Critically important: High Priority	Aminoglycosides	aminoglycoside	Ground water(Gallusquelle) [Germany, Mar2012-Jun2013 (Stange)];Rivers Rhine, Danube [Germany (and Australia), not reported (Stoll)];River Weser [Germany, Jul-Sep2018 (Song)]
Water body	Critically important: High Priority	Aminopenicillin with beta-lactamase inhibitors	amoxicillin/clavulanic acid	Danube, Glan, Inn, Traun [Austria, 2016 (Lepuschitz)]
Water body	Critically important: High Priority	Carbapenems	carbapenem	River lower Lahn [Germany, Oct2011-Dec2012 (Herrig)]
Water body	Critically important: High Priority	Glycylcyclines	glycopeptide	Rivers Rhine, Danube [Germany (and Australia), not reported (Stoll)]
Water body	Critically important: High Priority	Macrolides	erythromycin	Danube, Glan, Inn, Traun [Austria, 2016 (Lepuschitz)]
Water body	Critically important: High Priority	Macrolides	macrolide	Ground water(Gallusquelle) [Germany, Mar2012-Jun2013 (Stange)];Rivers Rhine, Danube [Germany (and Australia), not reported (Stoll)]
Water body	Critically important: High Priority	Phosphonics	fosfomycin	Rural, urban water [Switzerland, 2012-2019 (Biggel)];Drinking water [Germany, Aug2018-Jun2019 (Voigt)]
Water body	Highly important	Amphenicols	chloramphenicol	Rivers Rhine, Danube [Germany (and Australia), not reported (Stoll)]
Water body	Highly important	Cephalosporins	cephalosporin	Rivers, inland canals, streams [Switzerland, May-Aug2019

				(Bleichenbacher)];River lower Lahn [Germany, Oct2011-Dec2012 (Herrig)];River Weser [Germany, Jul-Sep2018 (Song)]
Water body	Highly important	First-generation cephalosporins	cefazolin	River Swist [Germany, not reported (Schreiber)]
Water body	Highly important	Penicillins	amoxicillin	River Rhine [Germany, Jun2006-Jun2007 (Stange)]
Water body	Highly important	Penicillins	ampicillin	River Mur [Austria, Oct2015-Jan2016 (Zarfel)]
Water body	Highly important	Penicillins	benzylpenicillin	Danube, Glan, Inn, Traun [Austria, 2016 (Lepuschitz)]
Water body	Highly important	Penicillins	oxacillin	River Swist [Germany, not reported (Schreiber)]
Water body	Highly important	Penicillins	penicillins	River lower Lahn [Germany, Oct2011-Dec2012 (Herrig)];River Weser [Germany, Jul-Sep2018 (Song)]
Water body	Highly important	Penicillins	piperacillin	River Swist [Germany, not reported (Schreiber)];Drinking water [Germany, Aug2018-Jun2019 (Voigt)];River Mur [Austria, Oct2015-Jan2016 (Zarfel)]
Water body	Highly important	Second-generation cephalosporins	cefoxitin	Danube, Glan, Inn, Traun [Austria, 2016 (Lepuschitz)]
Water body	Highly important	Second-generation cephalosporins	cefuroxime	River Mur [Austria, Oct2015-Jan2016 (Zarfel)]
Water body	Highly important	Sulfonamides	sulfonamide	Ground water(Gallusquelle) [Germany, Mar2012-Jun2013 (Stange)];Rivers Rhine, Danube [Germany (and Australia), not reported (Stoll)]
Water body	Highly important	Tetracyclines	tetracycline	River Rhine [Germany, Jun2006-Jun2007 (Stange)];Ground water(Gallusquelle) [Germany, Mar2012-Jun2013 (Stange)];Rivers Rhine, Danube [Germany (and Australia), not reported (Stoll)];River Mur [Austria, Oct2015-Jan2016 (Zarfel)]
Water body	Highly important	Trimethoprim	trimethoprim	Ground water(Gallusquelle) [Germany, Mar2012-Jun2013 (Stange)];Rivers Rhine, Danube [Germany (and Australia), not reported (Stoll)]
Water body	Highly important	Trimethoprim - sulfonamide combinations	trimethoprim /sulfamethoxazole	River Rhine [Germany, Jun2006-Jun2007 (Stange)];Drinking water [Germany, Aug2018-Jun2019 (Voigt)];River Weser [Germany, Jul-Sep2018 (Song)];River Mur [Austria, Oct2015-Jan2016 (Zarfel)]
Water body	not listed	Beta lactamases	beta-lactames	Rivers Rhine, Danube [Germany (and Australia), not reported (Stoll)]

Water body	not listed	Extended spectrum beta lactamases	ESBL	Rural, urban water [Switzerland, 2012-2019 (Biggel)]
Water, sediment	Critically important: Highest Priority	Fluoroquinolon es	ciprofloxacin	Water, soil [Austria, 2007-2009 (Linke)]
Water, sediment	Critically important: Highest Priority	Fluoroquinolon es	norfloxacin	Sediment(Lake Geneva) [Switzerland Democratic Republic of the Congo, India , May2012 (Devarajan)]
Water, sediment	Critically important: Highest Priority	Fluoroquinolon es	ofloxacin	Sediment(Lake Geneva) [Switzerland Democratic Republic of the Congo, India , May2012 (Devarajan)];Water, soil [Austria, 2007-2009 (Linke)]
Water, sediment	Critically important: Highest Priority	Fluoroquinolon es	qnrA	Sediment(Lake Geneva) [Switzerland, Aug 11 (Czekalski)]
Water, sediment	Critically important: Highest Priority	Fourth-generation cephalosporins	cefepime	Sediment(Lake Geneva) [Switzerland Democratic Republic of the Congo, India , May2012 (Devarajan)]
Water, sediment	Critically important: Highest Priority	Quinolones	nalidixic acid	Wastewater, sediment close to WWTP [Switzerland, not reported (Czekalski)]
Water, sediment	Critically important: Highest Priority	Third-generation cephalosporins	cefoperazone	Sediment(Lake Geneva) [Switzerland Democratic Republic of the Congo, India , May2012 (Devarajan)]
Water, sediment	Critically important: Highest Priority	Third-generation cephalosporins	cefotaxime	Water, soil [Austria, 2007-2009 (Linke)]
Water, sediment	Critically important: Highest Priority	Third-generation cephalosporins	ceftazidime	Sediment(Lake Geneva) [Switzerland Democratic Republic of the Congo, India , May2012 (Devarajan)]
Water, sediment	Critically important: Highest Priority	Third-generation cephalosporins	ceftriaxone	Water, soil [Austria, 2007-2009 (Linke)]
Water, sediment	Critically important: High Priority	Aminoglycosid es	kanamycin	Soil(Maize) [Austria, Aug-Sep2011 (Woegerbauer)];Soil(Potato) [Austria, Aug-Sep2011 (Woegerbauer)]
Water, sediment	Critically important: High Priority	Aminoglycosid es	nptII	Soil(Maize) [Austria, Aug-Sep2011 (Woegerbauer)];Soil(Potato) [Austria, Aug-Sep2011 (Woegerbauer)]
Water, sediment	Critically important: High Priority	Aminoglycosid es	nptIII	Soil(Maize) [Austria, Aug-Sep2011 (Woegerbauer)];Soil(Potato) [Austria, Aug-Sep2011 (Woegerbauer)]

Water, sediment	Critically important: High Priority	Aminoglycosides	streptomycin	Sediment(Lake Geneva) [Switzerland Democratic Republic of the Congo, India , May2012 (Devarajan)];Coastal water Baltic, North Sea [Germany, 2004–2012.2009–2014 (Bier)];Wastewater, sediment close to WWTP [Switzerland, not reported (Czekalski)]
Water, sediment	Critically important: High Priority	Carbapenems	imipenem	Sediment(Lake Geneva) [Switzerland Democratic Republic of the Congo, India , May2012 (Devarajan)];Coastal water Baltic, North Sea [Germany, 2004–2012.2009–2014 (Bier)]
Water, sediment	Critically important: High Priority	Carbapenems	meropenem	Coastal water Baltic, North Sea [Germany, 2004–2012.2009–2014 (Bier)]
Water, sediment	Critically important: High Priority	Macrolides	erythromycin	Water, soil [Austria, 2007-2009 (Linke)]
Water, sediment	Critically important: High Priority	Oxazolidinones	linezolid	Water, soil [Austria, 2007-2009 (Linke)]
Water, sediment	Highly important	Amphenicols	chloramphenicol	Wastewater, sediment close to WWTP [Switzerland, not reported (Czekalski)]
Water, sediment	Highly important	Cephalosporins	cephalosporin	WWTP-impacted water, soil [Germany, Feb-Jun2019 (Reichert)]
Water, sediment	Highly important	Penicillins	ampicillin	Coastal water Baltic, North Sea [Germany, 2004–2012.2009–2014 (Bier)];Wastewater, sediment close to WWTP [Switzerland, not reported (Czekalski)]
Water, sediment	Highly important	Penicillins	piperacillin	Sediment(Lake Geneva) [Switzerland Democratic Republic of the Congo, India , May2012 (Devarajan)]
Water, sediment	Highly important	Sulfonamides	sul1	Sediment(Lake Geneva) [Switzerland, Aug 11 (Czekalski)];Sediment(Lake Bret) [Switzerland, Sep 17 (Laffite)]
Water, sediment	Highly important	Sulfonamides	sul2	Sediment(Lake Geneva) [Switzerland, Aug 11 (Czekalski)];Sediment(Lake Bret) [Switzerland, Sep 17 (Laffite)]
Water, sediment	Highly important	Trimethoprim	trimethoprim	Coastal water Baltic, North Sea [Germany, 2004–2012.2009–2014 (Bier)]
Water, sediment	Highly important	Trimethoprim - sulfonamide combinations	trimethoprim /sulfamethoxazole	Wastewater, sediment close to WWTP [Switzerland, not reported (Czekalski)]
Water, sediment	not listed	Beta lactamases	beta-lactames	Sediment(Lake Geneva) [Switzerland, not reported (Thevenon)]
Water, sediment	not listed	Beta lactamases	blaCTX-M	Sediment(Lake Bret) [Switzerland, Sep 17 (Laffite)]

Water, sediment	not listed	Beta lactamases	blaSHV	Sediment(Lake Bret) [Switzerland, Sep 17 (Laffite)]
Water, sediment	not listed	Beta lactamases	blaTEM	Sediment(Lake Bret) [Switzerland, Sep 17 (Laffite)]
Wildlife	Critically important: Highest Priority	Fluoroquinolones	ciprofloxacin	Mouflons [Austria, Germany, Mar2012-Jan2016 (Loncaric)];Boars [Germany, 2016 (Plaza-Rodríguez)];Roe deer [Germany, 2017 (Plaza-Rodríguez)];Ducks, geese [Germany, 2019 (Plaza-Rodríguez)];Flies [Germany, Jul 16 (Wetzker)];Birds [Switzerland, May-Oct2018 (Zurfluh)]
Wildlife	Critically important: Highest Priority	Fluoroquinolones	enrofloxacin	Mouflons [Austria, Germany, Mar2012-Jan2016 (Loncaric)]
Wildlife	Critically important: Highest Priority	Fluoroquinolones	ofloxacin	Mouflons [Austria, Germany, Mar2012-Jan2016 (Loncaric)];Boars [Germany, 2016 (Plaza-Rodríguez)];Roe deer [Germany, 2017 (Plaza-Rodríguez)];Ducks, geese [Germany, 2019 (Plaza-Rodríguez)];Flies [Germany, Jul 16 (Wetzker)];Birds [Switzerland, May-Oct2018 (Zurfluh)]
Wildlife	Critically important: Highest Priority	Fourth-generation cephalosporins	cefquinome	Mouflons [Austria, Germany, Mar2012-Jan2016 (Loncaric)]
Wildlife	Critically important: Highest Priority	Polymyxins	colistin	Boars [Germany, 2016 (Plaza-Rodríguez)];Ducks, geese [Germany, 2019 (Plaza-Rodríguez)];Boars [Germany, Feb2015-Dec2016 (Reinhardt)]
Wildlife	Critically important: Highest Priority	Quinolones	nalidixic acid	Mouflons [Austria, Germany, Mar2012-Jan2016 (Loncaric)];Boars [Germany, 2016 (Plaza-Rodríguez)];Ducks, geese [Germany, 2019 (Plaza-Rodríguez)]
Wildlife	Critically important: Highest Priority	Third-generation cephalosporins	cefoperazone	Mouflons [Austria, Germany, Mar2012-Jan2016 (Loncaric)]
Wildlife	Critically important: Highest Priority	Third-generation cephalosporins	cefotaxime	Mouflons [Austria, Germany, Mar2012-Jan2016 (Loncaric)];Resident rooks [Austria, Mar2013 (Loncaric)];Boars [Germany, 2016 (Plaza-Rodríguez)];Roe deer [Germany, 2017 (Plaza-Rodríguez)];Ducks, geese [Germany, 2019 (Plaza-Rodríguez)]
Wildlife	Critically important: Highest Priority	Third-generation cephalosporins	ceftazidime	Boars [Germany, 2016 (Plaza-Rodríguez)];Roe deer [Germany, 2017 (Plaza-Rodríguez)];Ducks,

				geese [Germany, 2019 (Plaza-Rodríguez)]
Wildlife	Critically important: Highest Priority	Third-generation cephalosporins	ceftiofur	Mouflons [Austria, Germany, Mar2012-Jan2016 (Loncaric)]
Wildlife	Critically important: High Priority	Aminoglycosides	gentamicin	Mouflons [Austria, Germany, Mar2012-Jan2016 (Loncaric)];Boars [Germany, 2016 (Plaza-Rodríguez)];Roe deer [Germany, 2017 (Plaza-Rodríguez)];Ducks, geese [Germany, 2019 (Plaza-Rodríguez)];Birds [Switzerland, May-Oct2018 (Zurfluh)]
Wildlife	Critically important: High Priority	Aminoglycosides	kanamycin	Rodents [Germany, 2007-2008 (Guenther)];Birds [Switzerland, May-Oct2018 (Zurfluh)]
Wildlife	Critically important: High Priority	Aminoglycosides	neomycin	Mouflons [Austria, Germany, Mar2012-Jan2016 (Loncaric)]
Wildlife	Critically important: High Priority	Aminoglycosides	strA	Birds [Germany, 2011-2014 (Schaufler)]
Wildlife	Critically important: High Priority	Aminoglycosides	strB	Birds [Germany, 2011-2014 (Schaufler)]
Wildlife	Critically important: High Priority	Aminoglycosides	streptomycin	Rodents [Germany, 2007-2008 (Guenther)];Mouflons [Austria, Germany, Mar2012-Jan2016 (Loncaric)];Birds [Switzerland, May-Oct2018 (Zurfluh)]
Wildlife	Critically important: High Priority	Aminoglycosides	tobramycin	Resident rooks [Austria, Mar2013 (Loncaric)]
Wildlife	Critically important: High Priority	Aminopenicillin with beta-lactamase inhibitors	amoxicillin/clavulanic acid	Mouflons [Austria, Germany, Mar2012-Jan2016 (Loncaric)];Birds [Switzerland, May-Oct2018 (Zurfluh)]
Wildlife	Critically important: High Priority	Carboxypenicillins	ticarcillin	Rodents [Germany, 2007-2008 (Guenther)]
Wildlife	Critically important: High Priority	Macrolides	azithromycin	Boars [Germany, 2016 (Plaza-Rodríguez)];Roe deer [Germany, 2017 (Plaza-Rodríguez)];Ducks, geese [Germany, 2019 (Plaza-Rodríguez)];Birds [Switzerland, May-Oct2018 (Zurfluh)]
Wildlife	Highly important	Amphenicols	chloramphenicol	Mouflons [Austria, Germany, Mar2012-Jan2016 (Loncaric)];Migratory rooks [Austria, Mar2013 (Loncaric)];Boars [Germany, 2016 (Plaza-Rodríguez)]
Wildlife	Highly important	Amphenicols	florfenicol	Mouflons [Austria, Germany, Mar2012-Jan2016 (Loncaric)]
Wildlife	Highly important	First-generation cephalosporins	cefalotin	Mouflons [Austria, Germany, Mar2012-Jan2016 (Loncaric)]

Wildlife	Highly important	First-generation cephalosporins	cefazolin	Birds [Switzerland, May-Oct2018 (Zurfluh)]
Wildlife	Highly important	Penicillins	ampicillin	Rodents [Germany, 2007-2008 (Guenther)];Mouflons [Austria, Germany, Mar2012-Jan2016 (Loncaric)];Migratory rooks [Austria, Mar2013 (Loncaric)];Resident rooks [Austria, Mar2013 (Loncaric)];Boars [Germany, 2016 (Plaza-Rodríguez)];Roe deer [Germany, 2017 (Plaza-Rodríguez)];Ducks, geese [Germany, 2019 (Plaza-Rodríguez)]
Wildlife	Highly important	Penicillins	piperacillin	Migratory rooks [Austria, Mar2013 (Loncaric)];Resident rooks [Austria, Mar2013 (Loncaric)]
Wildlife	Highly important	Sulfonamides	sul1	Birds [Germany, 2011-2014 (Schaufler)]
Wildlife	Highly important	Sulfonamides	sul2	Birds [Germany, 2011-2014 (Schaufler)]
Wildlife	Highly important	Sulfonamides	sul3	Birds [Germany, 2011-2014 (Schaufler)]
Wildlife	Highly important	Tetracyclines	doxycycline	Mouflons [Austria, Germany, Mar2012-Jan2016 (Loncaric)]
Wildlife	Highly important	Tetracyclines	tetracycline	Rodents [Germany, 2007-2008 (Guenther)];Mouflons [Austria, Germany, Mar2012-Jan2016 (Loncaric)];Migratory rooks [Austria, Mar2013 (Loncaric)];Boars [Germany, 2016 (Plaza-Rodríguez)];Roe deer [Germany, 2017 (Plaza-Rodríguez)];Ducks, geese [Germany, 2019 (Plaza-Rodríguez)]
Wildlife	Highly important	Trimethoprim	trimethoprim	Boars [Germany, Feb2015-Dec2016 (Reinhardt)]
Wildlife	Highly important	Trimethoprim - sulfonamide combinations	trimethoprim /sulfamethox azole	Rodents [Germany, 2007-2008 (Guenther)];Mouflons [Austria, Germany, Mar2012-Jan2016 (Loncaric)];Migratory rooks [Austria, Mar2013 (Loncaric)];Boars [Germany, 2016 (Plaza-Rodríguez)];Roe deer [Germany, 2017 (Plaza-Rodríguez)];Ducks, geese [Germany, 2019 (Plaza-Rodríguez)];Flies [Germany, Jul 16 (Wetzker)];Birds [Switzerland, May-Oct2018 (Zurfluh)]
Wildlife	Important	Aminocyclitols	spectinomyci n	Rodents [Germany, 2007-2008 (Guenther)]
Wildlife	Important	Nitrofurantoin	nitrofurantoi n	Birds [Switzerland, May-Oct2018 (Zurfluh)]
Wildlife	not listed	Beta lactamases	ampC	Birds [Germany, 2011-2014 (Schaufler)]
Wildlife	not listed	Beta lactamases	blaCTX-M	Birds [Germany, 2011-2014 (Schaufler)]

Wildlife	not listed	Beta lactamases	blaCTX-M-1	Birds [Germany, 2011-2014 (Schaufler)]
Wildlife	not listed	Beta lactamases	blaCTX-M-15	Birds [Germany, 2011-2014 (Schaufler)]
Wildlife	not listed	Beta lactamases	blaOXA	Birds [Germany, 2011-2014 (Schaufler)]
Wildlife	not listed	Beta lactamases	blaOXA-1	Birds [Germany, 2011-2014 (Schaufler)]
Wildlife	not listed	Beta lactamases	blaTEM	Birds [Germany, 2011-2014 (Schaufler)]
Wildlife	not listed	Beta lactamases	blaTEM-1	Birds [Germany, 2011-2014 (Schaufler)]
Wildlife	not listed	Extended spectrum beta lactamases	ESBL	Flies [Germany, Jul 16 (Wetzker)]

2.5. Excluded studies

Studies excluded during the full-text screening (n = 33):

Excluded due to occurrence was not investigated (n = 9)

1. Falgenhauer L, Ghosh H, Guerra B, Yao Y, Fritzenwanker M, Fischer J, et al. Comparative genome analysis of IncHI2 VIM-1 carbapenemase-encoding plasmids of Escherichia coli and Salmonella enterica isolated from a livestock farm in Germany. Veterinary Microbiology. 2017;200:114-7.
2. Gallati C, Stephan R, Hächler H, Malorny B, Schroeter A, Nüesch-Inderbinen M. Characterization of salmonella enterica subsp. Enterica serovar 4,[5],12:I:- Clones isolated from human and other sources in Switzerland between 2007 and 2011. Foodborne Pathogens and Disease. 2013;10(6):549-54.
3. Hänel I, Müller E, Santamarina BG, Tomaso H, Hotzel H, Busch A. Antimicrobial Susceptibility and Genomic Analysis of Aliarcobacter cibarius and Aliarcobacter thereius, Two Rarely Detected Aliarcobacter Species. Frontiers in Cellular and Infection Microbiology. 2021;11.
4. Hindermann D, Gopinath G, Chase H, Negrete F, Althaus D, Zurfluh K, et al. Salmonella enterica serovar infantis from food and human infections, Switzerland, 2010-2015: Poultry-related multidrug resistant clones and an emerging ESBL producing clonal lineage. Frontiers in Microbiology. 2017;8(JUL).
5. Huehn S, Helmuth R, Bunge C, Guerra B, Junker E, Davies RH, et al. Characterization of pathogenic and resistant genome repertoire reveals two clonal lines in Salmonella enterica subsp. enterica serovar Paratyphi B (+)-tartrate positive. Foodborne Pathogens and Disease. 2009;6(4):431-43.
6. Schlüter A, Heuer H, Szczepanowski R, Forney LJ, Thomas CM, Pühler A, et al. The 64 508 bp IncP-1β antibiotic multiresistance plasmid pB10 isolated from a waste-water treatment plant provides evidence for recombination between members of different branches of the IncP-1β group. Microbiology (Reading). 2003;149(11):3139-53.
7. Sood S, Awal RP, Wink J, Mohr KI, Rohde M, Stadler M, et al. Aggregicoccus edonensis gen. nov., sp. nov., an unusually aggregating myxobacterium isolated from a soil sample. International Journal of Systematic and Evolutionary Microbiology. 2015;65(3):745-53.
8. Steinmann J, Mamat U, Abda EM, Kirchhoff L, Streit WR, Schaible UE, et al. Analysis of phylogenetic variation of Stenotrophomonas maltophilia reveals human-specific branches. Frontiers in Microbiology. 2018;9(APR).
9. Zurfluh K, Power KA, Klumpp J, Wang J, Fanning S, Stephan R. A novel Tn3-like composite transposon harboring blaVIM-1 in klebsiella pneumoniae spp. pneumoniae Isolated from River Water. Microbial Drug Resistance. 2015;21(1):43-9.

Excluded due to study designs: review, protocol, or abstract (n=4)

1. Klare I, Konstabel C, Badstübner D, Werner G, Witte W. Occurrence and spread of antibiotic resistances in Enterococcus faecium. International Journal of Food Microbiology. 2003;88(2/3):269-90.
2. Savin M, Parcina M, Schmoger S, Kreyenschmidt J, Käsbohrer A, Hammerl JA. Draft genome sequences of acinetobacter baumannii isolates recovered from sewage water from a poultry slaughterhouse in Germany. Microbiology Resource Announcements. 2019;8(28).
3. Wareth G, Neubauer H. The animal-foods-environment interface of Klebsiella pneumoniae in Germany: an observational study on pathogenicity, resistance development and the current situation. Veterinary Research. 2021;52(16).
4. Wareth G, Neubauer H, Sprague LD. Acinetobacter baumannii – a neglected pathogen in veterinary and environmental health in Germany. Veterinary Research Communications. 2019;43(1).

Excluded due to the sample not from environment (n = 8):

1. Eikmeyer FG, Rademacher A, Hanreich A, Hennig M, Jaenicke S, Maus I, et al. Detailed analysis of metagenome datasets obtained from biogas-producing microbial communities residing in

biogas reactors does not indicate the presence of putative pathogenic microorganisms. Biotechnology for Biofuels. 2013;6(49):(4 April 2013).
2. Mateus-Vargas RH, Atanassova V, Reich F, Klein G. Antimicrobial susceptibility and genetic characterization of Escherichia coli recovered from frozen game meat. Food microbiology. 2017;63:164-9.
3. Noll M, Kleta S, Al-Dahouk S. Antibiotic susceptibility of 259 Listeria monocytogenes strains isolated from food, food-processing plants and human samples in Germany. Journal of Infection and Public Health. 2018;11(4):572-7.
4. Odenthal S, Akineden Ö, Usleber E. Extended-spectrum β-lactamase producing Enterobacteriaceae in bulk tank milk from German dairy farms. International Journal of Food Microbiology. 2016;238:72-8.
5. Schages L, Lucassen R, Wichern F, Kalscheuer R, Bockmühl D. The household resistome: frequency of β-lactamases, class 1 integrons, and antibiotic-resistant bacteria in the domestic environment and their reduction during automated dishwashing and laundering. Applied and Environmental Microbiology. 2020;86(23).
6. Schaufler K, Bethe A, Lübke-Becker A, Ewers C, Kohn B, Wieler LH, et al. Putative connection between zoonotic multiresistant extended-spectrum beta-lactamase (ESBL)-producing escherichia coli in dog feces from a veterinary campus and clinical isolates from dogs. Infection Ecology and Epidemiology. 2015;5(1):1-5.
7. Schmidt JS, Kuster SP, Nigg A, Dazio V, Brilhante M, Rohrbach H, et al. Poor infection prevention and control standards are associated with environmental contamination with carbapenemase-producing Enterobacterales and other multidrug-resistant bacteria in Swiss companion animal clinics. Antimicrobial Resistance and Infection Control. 2020;9(1).
8. Spohr M, Rau J, Friedrich A, Klittich G, Fetsch A, Guerra B, et al. Methicillin-resistant Staphylococcus aureus (MRSA) in three dairy herds in southwest Germany. Zoonoses and Public Health. 2011;58(4):252-61.

Excluded due to the focus of the study was antibiotic pollution (n =1)

Rodriguez-Mozaz S, Vaz-Moreira I, Varela Della Giustina S, Llorca M, Barceló D, Schubert S, et al. Antibiotic residues in final effluents of European wastewater treatment plants and their impact on the aquatic environment. Environment International. 2020;140.

Excluded due to the study was an experiment (n = 1)

Schmitt H, Stoob K, Hamscher G, Smit E, Seinen W. Tetracyclines and tetracycline resistance in agricultural soils: Microcosm and field studies. Microbial Ecology. 2006;51(3):267-76.

Modelling of environmental concentrations (n = 1)

Menz J, Olsson O, Kümmerer K. Antibiotic residues in livestock manure: does the EU risk assessment sufficiently protect against microbial toxicity and selection of resistant bacteria in the environment? Journal of Hazardous Materials. 2019;379.

Excluded due to conducted in other country (n = 1)

Pedroso AA, Hurley-Bacon AL, Zedek AS, Kwan TW, Jordan APO, Avellaneda G, et al. Can probiotics improve the environmental microbiome and resistome of commercial poultry production? International Journal of Environmental Research and Public Health. 2013;10(10):4534-59.

Excluded because the results are not reported (n = 3)

1. Borowiak M, Baumann B, Fischer J, Thomas K, Deneke C, Hammerl JA, et al. Development of a Novel mcr-6 to mcr-9 Multiplex PCR and Assessment of mcr-1 to mcr-9 Occurrence in Colistin-Resistant Salmonella enterica Isolates From Environment, Feed, Animals and Food (2011–2018) in Germany. Frontiers in Microbiology. 2020;11.

2. Pärnänen KMM, Narciso-da-Rocha C, Kneis D, Berendonk TU, Cacace D, Thi Thuy D, et al. Antibiotic resistance in European wastewater treatment plants mirrors the pattern of clinical antibiotic resistance prevalence. Science Advances. 2019;5(3):eaau9124.
3. Wengenroth L, Berglund F, Blaak H, Chifiriuc MC, Flach CF, Pircalabioru GG, et al. Antibiotic resistance in Wastewater Treatment Plants and transmission risks for employees and residents: the concept of the AWARE study. Antibiotics. 2021;10(5).

Excluded due to study of direct surroundings of farm animals (n = 5)

1. Dahms C, Hübner NO, Cuny C, Kramer A. Occurrence of methicillin-resistant Staphylococcus aureus in farm workers and the livestock environment in Mecklenburg-Western Pomerania, Germany. Acta veterinaria Scandinavica. 2014;56:53.
2. Hille K, Roschanski N, Ruddat I, Woydt J, Hartmann M, Rösler U, et al. Investigation of potential risk factors for the occurrence of Escherichia coli isolates from German fattening pig farms harbouring the mcr-1 colistin–resistance gene. International Journal of Antimicrobial Agents. 2018;51(2):177-80.
3. Klein-Jöbstl D, Sofka D, Iwersen M, Drillich M, Hilbert F. Multilocus sequence typing and antimicrobial resistance of Campylobacter jejuni isolated from dairy calves in Austria. Frontiers in Microbiology. 2016;7(FEB).
4. Müller E, Abdel-Glil MY, Hotzel H, Hänel I, Tomaso H. Aliarcobacter butzleri from water poultry: Insights into antimicrobial resistance, virulence and heavy metal resistance. Genes. 2020;11(9):1-16.
5. Müller E, Hotzel H, Linde J, Hänel I, Tomaso H. Antimicrobial Resistance and in silico Virulence Profiling of Aliarcobacter butzleri Strains From German Water Poultry. Frontiers in Microbiology. 2020;11.
